# Oxytocin administration in neonates shapes hippocampal circuitry and restores social behavior in a mouse model of autism

**DOI:** 10.1038/s41380-021-01227-6

**Published:** 2021-07-21

**Authors:** Alessandra Bertoni, Fabienne Schaller, Roman Tyzio, Stephane Gaillard, Francesca Santini, Marion Xolin, Diabé Diabira, Radhika Vaidyanathan, Valery Matarazzo, Igor Medina, Elizabeth Hammock, Jinwei Zhang, Bice Chini, Jean-Luc Gaiarsa, Françoise Muscatelli

**Affiliations:** 1 Institut National de la Santé et de la Recherche Médicale (INSERM) UMR 1249, Institut de Neurobiologie de la Méditerranée (INMED), Institut Marseille Maladies Rares (MarMaRa), Aix-Marseille Université, Marseille, France; 2 Phenotype-expertise, Marseille, France; 3 Institute of Neuroscience, National Research Council (CNR), Vedano al Lambro, Italy. Department of Medical Biotechnology and Translational Medicine, Università degli Studi di Milano, Milan, Italy; 4 Institute of Neuroscience, National Research Council (CNR), Vedano al Lambro, Italy. NeuroMI Center for Neuroscience, University of Milano-Bicocca, Milan, Italy; 5 Florida State University, Tallahassee, FL USA; 6 Institute of Biomedical and Clinical Sciences, College of Medicine and Health, Hatherly Laboratories, University of Exeter, Exeter, UK

**Keywords:** Neuroscience, Diseases

## Abstract

Oxytocin is an important regulator of the social brain. In some animal models of autism, notably in *Magel2*^*tm1.1Mus*^-deficient mice, peripheral administration of oxytocin in infancy improves social behaviors until adulthood. However, neither the mechanisms responsible for social deficits nor the mechanisms by which such oxytocin administration has long-term effects are known. Here, we aimed to clarify these oxytocin-dependent mechanisms, focusing on social memory performance. Using in situ hybridization (RNAscope), we have established that *Magel2* and *oxytocin receptor* are co-expressed in the dentate gyrus and CA2/CA3 hippocampal regions involved in the circuitry underlying social memory. Then, we have shown that *Magel2*^*tm1.1Mus*^*-*deficient mice, evaluated in a three-chamber test, present a deficit in social memory. Next, in hippocampus, we conducted neuroanatomical and functional studies using immunostaining, oxytocin-binding experiments, ex vivo electrophysiological recordings, calcium imaging and biochemical studies. We demonstrated: an increase of the GABAergic activity of CA3-pyramidal cells associated with an increase in the quantity of oxytocin receptors and of somatostatin interneurons in both DG and CA2/CA3 regions. We also revealed a delay in the GABAergic development sequence in *Magel2*^*tm1.1Mus*^-deficient pups, linked to phosphorylation modifications of KCC2. Above all, we demonstrated the positive effects of subcutaneous administration of oxytocin in the mutant neonates, restoring hippocampal alterations and social memory at adulthood. Although clinical trials are debated, this study highlights the mechanisms by which peripheral oxytocin administration in neonates impacts the brain and demonstrates the therapeutic value of oxytocin to treat infants with autism spectrum disorders.

## Introduction

The nonapeptide oxytocin (OT) and its signaling pathway, the OT-system, are important regulators in the development of the social brain, suggesting that OT plays a role in both childhood and adult neuropsychiatric disorders characterized by social cognition impairment [1]. The OT-system is altered in several animal models of neurodevelopmental disorders [2, 3]. Indeed, knockout (KO) mouse models of *oxytocin* [4, 5], *oxytocin receptor* (*Oxtr*) [6–8], or *ADP-ribosyl cyclase* (*Cd38*) [9, 10] genes show changes in social behavior reminiscent of autism spectrum disorders (ASD). Additionally, several rodent models of ASD due either to the inactivation of genes such as *Fmr1, Cntnap2, Magel2, Oprm1, Shank3*, *Nlgn-3*, or environmental valproic acid exposure (VPA), exhibit indirect impairment of the brain OT-system [2]. *MAGEL2* is a gene that is involved in Prader–Willi (PWS) [11] and Schaaf-Yang (SYS) syndromes [12] and is classified as one of the highest relevant gene to ASD risk (SFARI gene scoring), indeed 75–80% of SYS patients meet the formal clinical diagnostic criteria of ASD [13, 14]. Both of these genetic neurodevelopmental disorders have in common autistic features with alterations in social behavior and deficits in cognition that persist over the lifespan [15]. *Magel2*^*tm1.1Mus*^*-*deficient mouse model is a pertinent model for both syndromes [15], mimicking alterations in social behavior and learning abilities in adulthood [16, 17]. *Magel2* is expressed in the developing hypothalamus until adulthood and *Magel2*^*tm1.1Mus*^*-*KO neonates display a deficiency of several hypothalamic neuropeptides, particularly OT [18]. Daily administration of OT in *Magel2-*KO neonates during the first week of life improves social behavior and learning abilities beyond the treatment period lasting into adulthood [16]. Comparable long-term effects have also been reported in other genetic rodent models such as the VPA-induced rat model [19], the *Cntnap2*, *Fmr1* KO mice [20, 21], and following maternal separation [22]. However, the neurobiological alterations involving the OT-system and responsible for social behavior deficits in these models are not known. Similarly, the mechanisms by which OT-treatment in infancy exerts its long-lasting beneficial effects, remain mysterious.

At adulthood, OT is thought toregulate aspects of social behavior via interactions with OXTRs in a number of key brain regions [23]. Social recognition memory deficit in adulthood is a robust phenotype, present in all the models with an alteration of the OT-system [24]. With regard to social memory, a critical role has been ascribed to hippocampal OXTR expression in the anterior hilar dentate gyrus (aDG) and anterior CA2/CA3 distal regions (aCA2/CA3d) [25–28]. In the aCA2/CA3d region, OXTRs are expressed in glutamatergic pyramidal neurons and in GABAergic interneurons, which account for over 90% of OXTR positive cells in the hippocampus [29]. Notably, both types of neuron are necessary for the formation of stronger synapses that mediate long-term potentiation and social memory [25, 26, 30].

In the first two postnatal weeks, OT neuron projections develop and the expression of OXTRs is extremely dynamic followed by a decreased expression thereafter [31, 32]. However, during this developmental period, the mechanisms by which the OT-system shapes various behaviors are little studied. One study reports that OT is involved in dendritic and synaptic refinement in immature hippocampal glutamatergic neurons [33].

Five clinical trials (phase 1 or 2) of OT administration in patients with PWS have been conducted, yielding positive or no effects, with no adverse effects reported [34]. However, the intervention design of each of the studies is rather exploratory, using different timings, durations, and doses of OT, since we do not yet understand clearly how OT works. Based on our previous preclinical studies [16, 18], a phase 1/2 clinical trial with OT-treatment of infants with PWS significantly improves early feeding and “social skills” [35], supporting the translational relevance of our study. More research is needed to demonstrate and validate our hypothesis that the administration of OT in early infancy might be the most beneficial treatment for PWS/SYS. Thus, to build a strong scientific rationale for clinical intervention, it is necessary to elucidate the PWS/SYS neuronal alterations and the mechanisms underlying the long-lasting effects of OT-administration in neonates.

Here, we aimed to clarify the physiological and cellular mechanisms related to the OT-system that are disturbed in *Magel2*^*tm1.1Mus*^*-*deficient mice and those responsible for the long-term rescue effects following OT peripheral administration in pups. We focused our study on the deficit of social memory, a robust phenotype linked to the OT-system.

## Materials and methods

### Animals and primary hippocampal cultures

*Magel2*^*tm1.1Mus*^+/+ (WT) and *Magel2*^*tm1.1Mus*^−/− (*Magel2-*KO) mice were maintained on a C57BL/6J genetic background. Experimental protocols were approved by the institutional Ethical Committee guidelines for animal research with the accreditation no. B13-055-19 from the French Ministry of Agriculture. *Magel2*-deficient mice were generated as previously published [18]. Embryonic day 18 dissociated hippocampal neurons were obtained from timed pregnant mice. See Supplementary Information for details.

### Oxytocin treatment

WT and *Magel2-*KO pups were removed from their mother, placed on a heating pad, given a subcutaneous (s.c.) injection and quickly returned to the mother. The solutions injected were isotonic saline (10 µl) for control mice and 2 µg of OT (Phoenix Pharmaceuticals Inc., cat #051-01) diluted in isotonic saline (10 µl) for treated mice.

### Behavior

The effects of *Magel2* deletion and OT-treatment were evaluated on social behavior, locomotor and vertical activity, anxiety-like behavior and nonsocial memory. For detailed procedures, see Supplementary Information.

### Calcium imaging recordings

Calcium imaging experiments were carried out as previously reported [36] and are described in Supplementary Information section.

### Electrophysiological recordings and morphological analysis

#### Whole-cell patch clamp

Spontaneous and miniature synaptic activity was recorded in voltage-clamp mode on P20–P25 CA3-pyramidal neurons. The morphology of recorded aCA3d neurons was defined by adding biocytin in the recording solution and performing Neurolucida reconstruction followed by a Sholl analysis. See Supplementary Information, for details.

*Single GABA*_*A*_ *channel recordings* were performed on hippocampal CA3-pyramidal neurons at P1, P7, and P15 in cell-attached configuration, as described in Supplementary Information.

### Immunohistochemistry and quantification

Immunostaining was carried out on 50 μm-thick coronal sections following standard procedure, as described in Supplementary Information.

### OT-binding assay

Adult WT and mutant mice were killed and non-perfused mouse brain were frozen in −25 °C isopentane and stored at −80 °C until cut. 14 µm-thick brain slices were cut using a cryostat (Frigocut-2700, Reichert-Jung) and collected on chromallume-coated slides and stored at −80 °C until use. Localization of OT-binding sites was performed by autoradiography as detailed in Supplementary Information.

### Chromogenic in situ hybridization

Fresh-frozen brains from WT mice at P7 and P28 were sectioned in a cryostat in the coronal plane at 20 μm thickness and mounted on Superfrost Plus slides and stored at −80 °C. RNA detection was performed on tissue sections using RNAscope 2.5HD Duplex Assay (Cat #322430, Advanced Cell Diagnostics (ACD), Hayward, CA) as detailed in Supplementary Information.

### Western blot

Western blotting experiments were performed on hippocampal tissue and specific bands were visualized with secondary HRP-conjugated antibodies using ChemiDoc™ Imaging Systems (Bio-Rad). The relative intensities of immunoblot bands were determined by densitometry with ImageJ software. See Supplementary Information for details.

### Statistical analysis

Statistical analyses were performed using GraphPad Prism (GraphPad Software, Prism 8.0 software, Inc, La Jolla, CA, USA). All the statistical analyses are reported in a specific file. For details, see Supplementary Information.

## Results

### aDG and aCA2/CA3d regions co-express *Magel2* and *Oxtr* transcripts

**Fig. 1:**
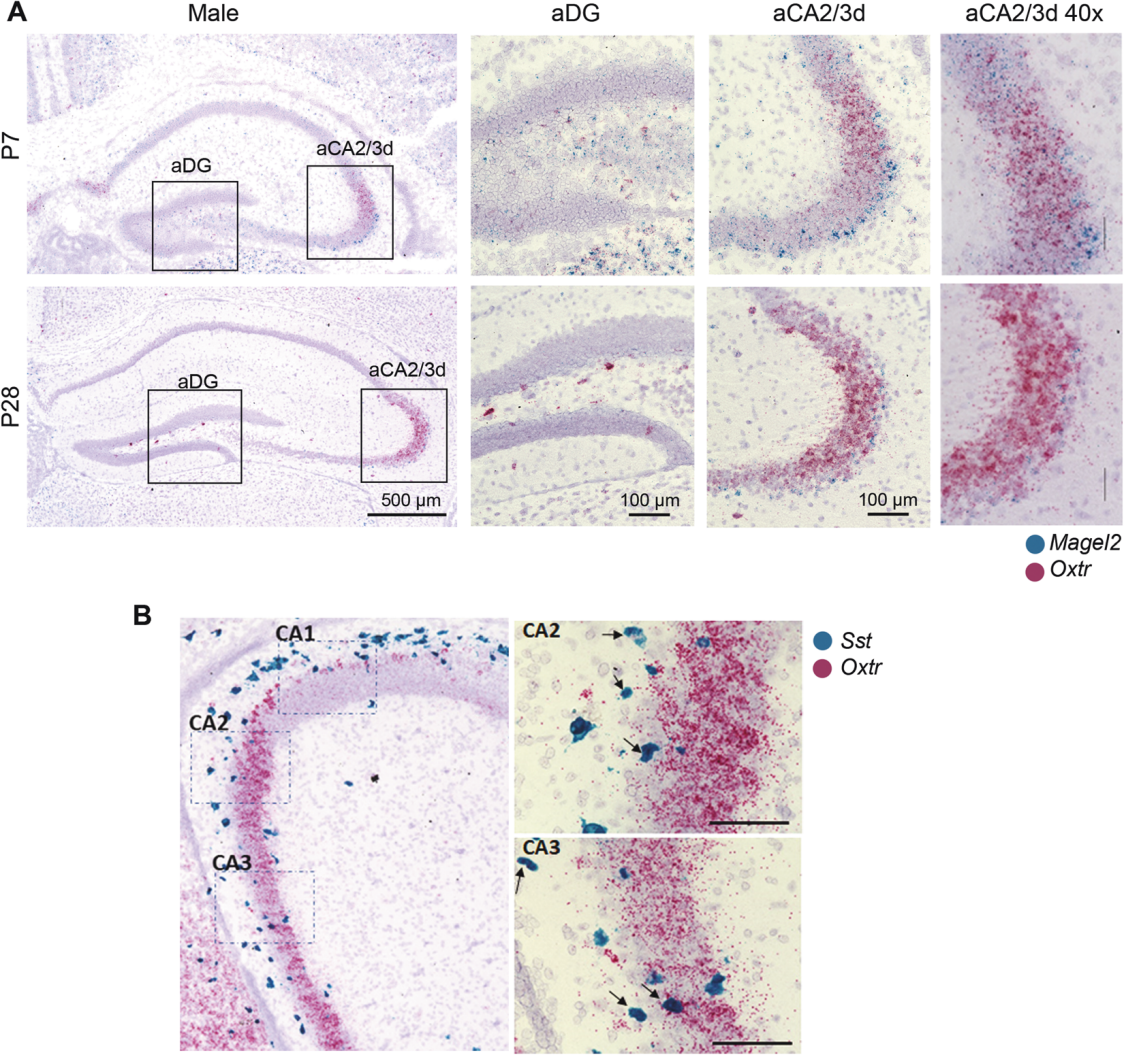
Expression of *Magel2* and *Oxytocin receptor* (*Oxtr*) transcripts in hippocampus of wild-type male mice at P7 and P28. **A** Representative image obtained by RNAscope technology showing the respective localization of *Magel2* (blue) and *Oxtr* (pink) transcripts in dentate gyrus (DG) and aCA2/CA3d region of hippocampus. **B** Representative image obtained by RNAscope technique showing the respective localization of *Sst* (blue) and *Oxtr* (pink) transcripts in the aCA2/CA3 region from hippocampal slices of WT male pups at P10. Arrows indicate co-localization of both transcripts in the same cell. Scale bar: 100 µm.

*Magel2* is known to be highly expressed in hypothalamus, while its expression in hippocampal regions is less well characterized. Taking into account the developmental and dynamic expression of *Oxtr* [31, 32], we looked at the expression of *Magel2* and *Oxtr* transcripts in the anterior hippocampus at P7 and P28, using RNAscope technique. At P7, we detected *Oxtr* and *Magel2* mRNAs in the aCA2/CA3d region with *Magel2* more expressed in the deep layer of the *stratum pyramidale* (Fig. 1A). At P28, the level of *Magel2* transcripts was reduced but still present in the deep layer of aCA2/CA3d region and *Oxtr* transcripts were also strongly expressed in pyramidal cells. Expression of *Magel2* and *Oxtr* was also detected in few cells of the *stratum oriens* and *stratum radiatum* where co-expression can be observed. In the DG, an expression of *Oxtr* and *Magel2* was detected in the hilus, with co-localization of both transcripts. Then, in parallel, we extracted data from public RNAseq data libraries obtained in adult mice (Allen brain; Linnarson lab: http://celltypes.brain-map.org/rnaseq/mouse/cortex-and-hippocampus*;* http://mousebrain.org/genesearch.html*)*. It appears that *Oxtr* and *Magel2* are co-expressed in CA3 excitatory neurons (expressing CCK) and also in several interneuron subpopulations expressing somatostatin (SST). Indeed, we observed a co-expression of *Oxtr* and *Sst* mRNAs in hippocampus (Fig. 1B).

Given the role of OT and this hippocampal region in social memory, we tested the hypothesis that there is a social memory deficit of *Magel2-*KO mice.

### Deficit of social memory in *Magel2-*KO males is rescued by neonatal OT-treatment

In these, as well as in all following neonatal treatment experiments, WT and *Magel2-*KO pups were naïve or treated over a period of 7 days (starting at birth) with a total of four subcutaneous administrations of physiological saline (“vehicle”) or oxytocin (2 µg or ~1 mg/Kg over the 7 days; “OT-treatment” or “+OT”), one single administration per day being performed on alternate days (Postnatal day P0, P2, P4, and P6).

**Fig. 2:**
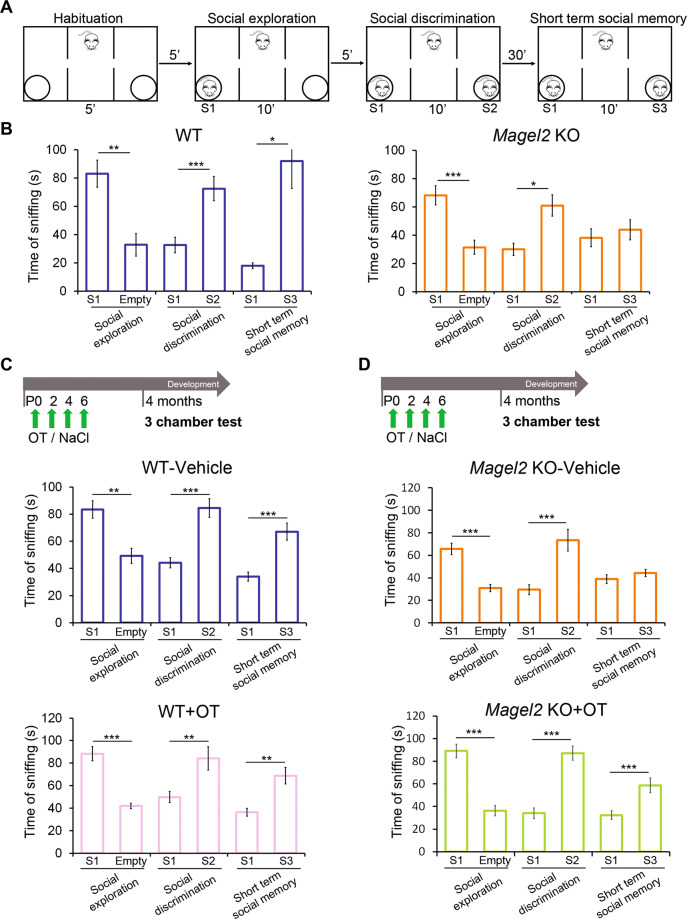
Social behavior in the three-chamber test of adult male WT versus *Magel2* KO and adult male WT or *Magel2* KO vehicle-treated or OT-treated as neonates. **A** Paradigm of the three-chamber test. Sniffing time between mice is measured in each test. **B** WT males (*N* = 12) show normal behavior in all the steps of the test; *Magel2* KO males (*N* = 9) show a significant impairment in short-term social memory. **C** WT mice were treated in the first week of life with vehicle or OT and then tested at 4 months. WT mice treated with vehicle (*N* = 18) or treated with OT (*N* = 10) have similar profiles with significant differences in each step of the test. **D** *Magel2* KO mice were treated in the first week of life with vehicle or OT and then tested at 4 months. *Magel2* KO-vehicle (*N* = 19) mice show a significant difference in the social exploration and social discrimination, but, in short-term social memory, they do not show a higher sniffing time with the novel mouse. OT-treated *Magel2* KO (*N* = 19) mice present significant differences in each step of the test. Data represented in histograms report the interaction time (time of sniffing in seconds) as mean ± SEM. Paired *t* test. *P < 0.05; **P < 0.01; ***P < 0.001. Statistical analysis is reported in Supplementary Table 1.

At adulthood, we investigated social behavior using the three-chamber test in order to assess social exploration (sociability), the preference for social novelty (social discrimination) and social memory (short-term social memory) (Fig. 2A). Adult *Magel2-*KO males showed levels of sociability and social discrimination similar to WT males but exhibited a significant deficit in social memory (Fig. 2B, Supplementary Fig. 1).

The effects of neonatal OT or vehicle treatment were first evaluated in WT male adults in the three-chamber test. We found that neither treatment had any measurable effect on sociability, social discrimination, or social memory: the amount of time spent sniffing in different compartments was similar to that recorded in untreated WT males (Fig. 2B, C). Furthermore, no significant effects of neonatal OT-treatment in WT animals were detected in widely used assays to test object recognition and social behavior (Supplementary Fig. 2A, B), motor activity (Supplementary Fig. 2B) and anxiety-like behaviors (Supplementary Fig. 2B, C).

Then, vehicle and OT-treatment were administered in *Magel2*-KO. *Magel2-*KO vehicle-treated males presented a social memory deficit similar to untreated *Magel2-*KO males (Fig. 2B, D). However, this deficit was rescued by neonatal OT-treatment (Fig. 2D). Sociability and social discrimination indices were not affected by vehicle or OT-treatment (Supplementary Fig. 1).

Cohorts of WT females untreated or treated with vehicle or OT during infancy were also tested at adulthood (excluding animals in the estrus phase). In the three-chamber test (Supplementary Fig. 3A), females preferred to interact with a mouse versus an empty compartment (sociability) (Supplementary Fig. 3B). However, in the social discrimination test, a less obvious preference to interact with a novel female compared with a familiar was observed (*P* values of 0.03 or 0.06) (Supplementary Fig. 3B, C) as previously reported [37]. Finally, all females failed in the short-term social memory test (Supplementary Fig. 3B, C). OT-treatment in the first week of life improved the level of sociability and of social discrimination showing a significant increase of sniffing time towards a conspecific compared with the vehicle or nontreated females but female mice still failed in the short-term social memory task (Supplementary Fig. 3C). Using the same battery of tests as in males, we also showed no effects of neonatal OT-treatment of WT females in anxiety-like behavior or novel object recognition but females treated with OT show significantly reduced distance moved in an open field (Supplementary Fig. 2). Overall, as this study aimed to investigate OT-dependent social memory mechanisms in mutant compared with wild-type mice, in absence of a female positive control group we decided to limit further study of mechanisms to males only.

Thus, the loss of *Magel2* causes a deficit in social memory in male *Magel2-*KO adults. This deficit was rescued by a neonatal OT-treatment. Due to the robust effect observed on social memory, we focused our subsequent investigations on the hippocampal region, previously shown to be specifically involved in OT-mediated effects on social memory [25, 26]. Neurons expressing the OXTRs in the CA2/CA3d and DG regions of the anterior hippocampus are involved in social memory [25, 26] therefore we tested the hypothesis that those regions are involved in the social memory deficit of *Magel2*-KO mice.

### Social memory test activates aDG and aCA2/CA3d in WT and *Magel2-*KO mice

**Fig. 3:**
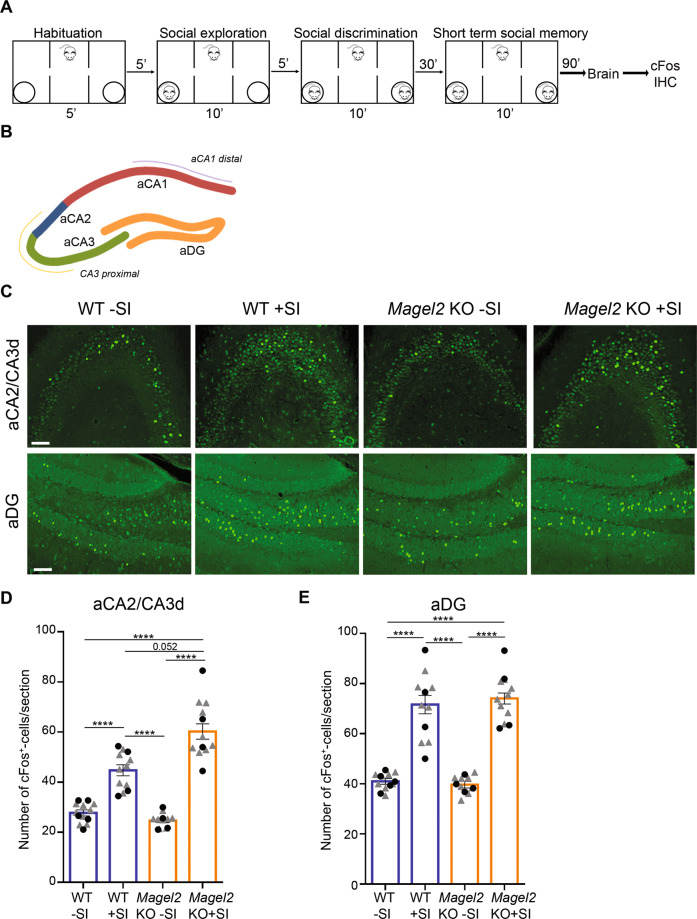
cFos activity in aCA2/CA3d and aDG regions of *Magel2* KO and WT adult male mice following the social memory task in the three-chamber test. **A** Paradigm of the three-chamber test (+SI) followed 90 min later by dissection of the brains and immunohistochemistry experiments. Control mice (−SI) were not tested in the three-chamber test. **B**, **C** cFos immunolabeling on coronal brain sections in the aCA2/CA3d and aDG regions as indicated in (**B**) of WT-SI, *Magel2* KO-SI, WT + SI and *Magel2* KO + SI mice (**C**). **D**, **E** Quantification of cFos+ cells/section in WT-SI (*n* = 40, *N* = 5), WT + SI (*n* = 31, *N* = 4), *Magel2* KO − SI (*n* = 32, *N* = 4) and *Magel2* KO + SI (*n* = 30, *N* = 4) in the aCA2/CA3d region (**D**) and in the aDG region (**E**). N number of animals, n number of sections. Scale bar: 500 µm (**B**); 100 µm (**C**). Data represented in histograms report the number of cFos+ cells by sections (6–8 sections/animal) as mean ± SEM for the genotype and treatment, with single gray data point (triangle) showing the average for each section level among all individuals of each group (since sections are repeated measures) and single black data point (circle) showing the mean value (of all sections) for each animal. Mixed model with repeated measurements + Tukey’s post hoc test, ***P < 0.001. Statistical analysis is reported in Supplementary Table 2.

WT and *Magel2-*KO mice were sacrificed 90 min after the end of social memory test (+SI, for Social Interactions) or without being tested (−SI) and their brains examined for cFos immunolabeling, a marker of neuronal activity, in the aDG and aCA2/CA3d regions (Fig. 3A, B). WT-SI and *Magel2-*KO-SI mice showed a similar quantity of cFos positive cells in both regions. In the aCA2/CA3d region, WT + SI versus WT − SI (Fig. 3C, D) showed a significant increase (×1.8) in the number of cFos+ cells; an increase (×2.2) was also observed in *Magel2-*KO + SI mice compared with *Magel2-*KO − SI (Fig. 3C, D), notably a tendency (*P* = 0.052) with an increase (×1.2) of cFos activated cells was observed in *Magel2-*KO + SI compared with WT + SI (Fig. 3D). In the aDG, mainly in the hilus and *stratum granulare*, a significant increase of ~60% of cFos+ cells was observed in both WT + SI and *Magel2-*KO + SI compared with untested (−SI) mice (Fig. 3C, E). Overall, these data confirm a strong activation of neurons in aDG and aCA2/CA3d regions following the social memory test in both WT and *Magel2-*KO mice, with an increased activation in the aCA2/CA3d *Magel2-*KO region.

### High levels of OT-binding sites in *Magel2-*KO hippocampi are reduced by neonatal OT-treatment

**Fig. 4:**
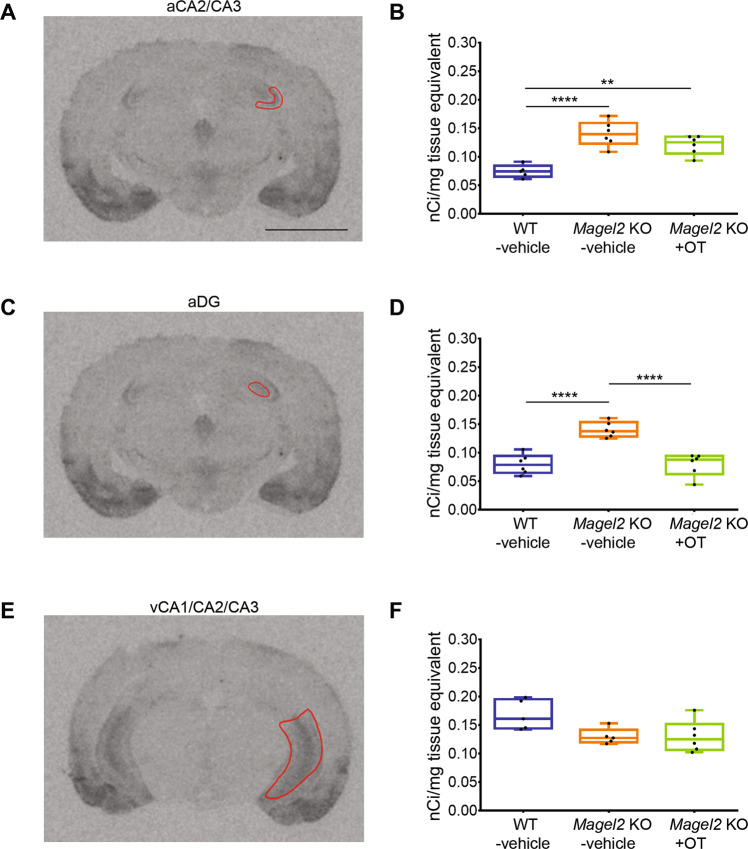
Quantification of OT-binding sites by brain autoradiography in the adult hippocampus of *Magel2* KO male mice treated with OT or vehicle versus WT-vehicle male mice. **A**, **C**, **E** Representative sections of autoradiographic labeling of OT-binding sites displayed in grayscale, showing the regions of interest (ROI) selected for analysis: (**A**) anterior CA2/CA3 (aCA2/CA3), (**C**) dentate gyrus (aDG), and (**E**) ventral CA1/CA2/CA3 (vCA1/CA2/CA3) regions of hippocampus. **B**, **D**, **F** Quantification of OT-binding sites expressed as nCi/mg of tissue equivalent in (**B**) anterior CA2/CA3, (**D**) dentate gyrus, and (**F**) ventral CA1/CA2/CA3 regions of hippocampus. Histograms report median (Q2) and quartiles (Q1, Q3). OT-binding sites in nCi/mg of tissue equivalent. Three mice and six hippocampi have been analyzed for each group. Data represented in box-plots report the quantity of radiolabeling by hippocampus, with scattered plots that show individual data points. One-way ANOVA + Bonferroni post hoc test, **P < 0.01; ****P < 0.0001. Scale Bar: 3 mm. Statistical analysis is reported in Supplementary Table 3.

We then looked at the distribution of OT-binding sites, reflecting the presence of functional OXTRs, in *Magel2-*KO-vehicle or *Magel2-*KO + OT compared with WT-vehicle hippocampi by autoradiography (Fig. 4). We observed a significant increase of OT-binding sites in the aCA2/CA3d (100%, Fig. 4A, B) and aDG (80%, Fig. 4C, D) regions but not in the ventral region (Fig. 4E, F) of *Magel2*-KO mice compared to WT. In *Magel2-*KO + OT we observed a normalization of the amount of OT-binding sites in the aDG (Fig. 4C, D); this amount is also decreased in the aCA2/CA3d region but remains high compared to the WT (Fig. 4A, B). This binding study indicates subregion-specific modulation of OXTRs in the hippocampus of *Magel2*-KO compared with WT. We then wondered if this specific effect could be linked to changes in neuronal subpopulations in these subregions.

### An increased number of SST+ neurons in the aCA2/CA3d and aDG regions of *Magel2-*KO adult mice is normalized by neonatal OT-treatment

**Fig. 5:**
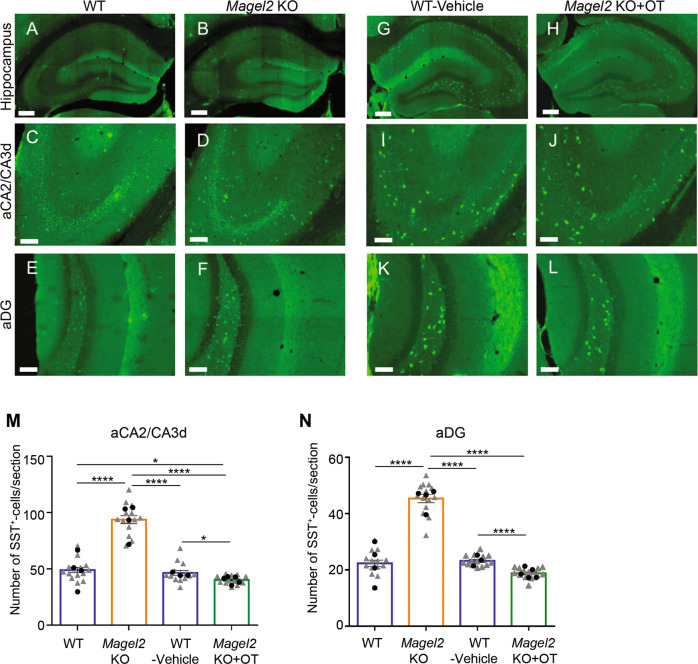
Quantification of somatostatin (SST) immunopositive cells in the anterior hippocampus region of adult *Magel2* KO, WT, *Magel2* KO treated with OT and WT-vehicle male mice. **A–L** Immunolabeling on coronal hippocampal sections at adulthood in WT (**A**, **C**, **E**) and *Magel2* KO (**B**, **D**, **F**), and in WT-vehicle (**G**, **I**, **K**) and *Magel2* KO + OT (**H**, **J**, **L**) with a magnification in the aCA2/CA3d region (**C**, **D**, **I**, **J**) and in the DG region (**E**, **F**, **K**, **L**) in which the SST+ cells are counted. **M**, **N** Number of SST+ cells by section in both aCA2/CA3d (**M**) and aDG (**N**) and comparing WT (*N* = 4, *n* = 56), *Magel2* KO (*N* = 4, *n* = 55), WT-vehicle (*N* = 3, *n* = 36), and *Magel2* KO + OT (*N* = 5, *n* = 64) mice. N, number of animals; n, number of sections/hippocampus. Data represented in histograms report the number of SST+ cells by section as mean ± SEM, with single gray data point (triangle) showing the average for each section level among all individuals of each group (since sections are repeated measures) and single black data point (circle) showing the mean value (of all sections) for each animal. Mixed model with repeated measurements + Tukey’s post hoc test. **P < 0.01; ***P < 0.001. Scale bar (**A**–**H**): 500 µm; (**C**–**L**): 100 µm. Statistical analysis is reported in Supplementary Table 4.

In the anterior adult hippocampus, OXTRs are expressed in pyramidal cells of aCA2/CA3d region and mainly in SST and/or PV interneurons of aCA2/CA3d and aDG [25, 29]. In *Magel2-*KO adult mice, the number of SST+ cells was significantly higher than in WT mice in both aCA2/CA3d (60% more) and aDG (80% more) regions (Fig. 5A–F, M, N). After a neonatal OT-treatment in *Magel2-*KO, the number of SST+ cells was slightly but significantly decreased in both aCA2/CA3d (10% less) and DG regions (17% less) compared with WT-vehicle mice (Fig. 5G–L, M, N); OT-treatment of *Magel2-*KO pups has significantly impacted the number of SST+ neurons at adulthood. PV+ cells were equally abundant in both genotypes (Supplementary Fig. 4). We then expected that the change in the number of SST+ cells had consequences in the alteration of the excitation/inhibition (E/I) balance, an electrophysiological feature frequently associated with multiple neurodevelopmental disorders.

### Neonatal OT-treatment normalizes the increased GABAergic activity in *Magel2-*KO aCA2/CA3d neurons but reduces the glutamatergic activity in both mutant and WT mice

**Fig. 6:**
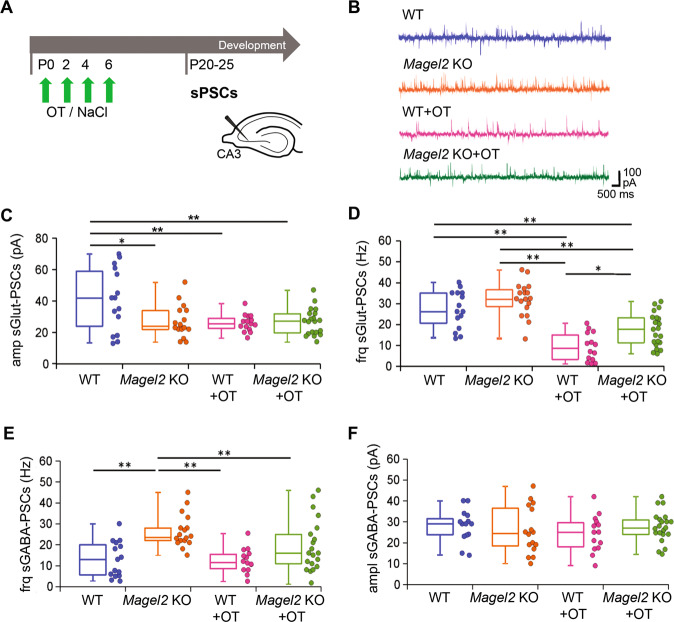
Spontaneous Glutamatergic and GABAergic synaptic activity of CA3-pyramidal neurons in the anterior hippocampus region of *Magel2* KO mice versus WT juvenile mice with or without OT-treatment as neonates. **A** Paradigm of the test. WT or *Magel2 KO* mice were either not injected or were injected with OT in the first week of life. At P20–25, neurons were recorded in brain slices. **B** Examples of whole-cell recordings performed at a holding potential of −45 mV for each genotype or treatment. The glutamatergic synaptic currents are inwards and the GABAergic synaptic currents are outwards. **C**, **D** Values in the different genotypes and treatment of the Glut-sPSCs amplitude (**C**) and frequency (**D**). **E**, **F** Values in the different genotypes and treatment of the GABA-sPSCs frequency (**E**) and amplitude (**F**). *Magel2* KO (*N* = 7, *n* = 16), WT (*N* = 7, *n* = 15), *Magel2* KO + OT, (*N* = 5, *n* = 21) and WT + OT (*N* = 4, *n* = 15) have been analyzed, with N number of mice and n number of recorded cells. Data represented in box-plots report the different values of recorded cells with mean ± SEM, with scattered plots showing individual data points. One-way ANOVA + Tukey post hoc test. *P < 0.05; **P < 0.01. Statistical analysis is reported in Supplementary Table 5.

Hippocampal brain slices of WT, *Magel2-*KO, WT + OT and *Magel2-*KO + OT male mice were analyzed using whole-cell patch clamp to record the activity of aCA2/CA3d pyramidal neurons (Fig. 6A). Spontaneous activities analysis (Fig. 6B) revealed a reduced amplitude of postsynaptic glutamatergic currents (sGlut-PSCs) in *Magel2-*KO as compared to WT mice (×1.7 less, Fig. 6C), while the frequency of sGlut-PSCs was not changed (Fig. 6D). The same *Magel2-*KO neurons presented a significant increase in GABAergic (sGABA-PSCs) frequency (×1.8, Fig. 6E), while the amplitude of sGABA-PSCs was similar to that of WT (Fig. 6F). Patch clamp recordings of the glutamatergic and GABAergic miniature currents (mGlut-PSCs and mGABA-PSCs, respectively) showed a significant reduction in amplitude of mGlut-PSCs, with no change in their frequency, in *Magel2-*KO neurons compared to WT (×1.4 less, Supplementary Fig. 5B, C), but no differences in frequencies and amplitudes of mGABA-PSCs (Supplementary Fig. 5D, E). An abnormal dendritic morphology of the pyramidal neurons could be associated with the alterations of the neuronal activities. However, we showed that dendritic morphology of *Magel2-*KO and WT CA3-pyramidal neurons were similar (Supplementary Fig. 6). Altogether, those results show that, in *Magel2-*KO pyramidal neurons of aCA3d, there is a significant increase in the GABA/Glutamate ratio with no change in their neuronal morphology.

We next investigated the effects of OT-treatment on the GABA/Glutamate balance in WT and *Magel2-*KO neurons. Quite unexpectedly, the frequency (×3 less) and amplitude (×1.7 less) of sGlut-PSCs were significantly reduced in WT + OT mice compared with WT (Fig. 6C, D). In *Magel2-*KO mutants, OT-treatment did not modify the amplitude but reduced the frequency (×2.9 less) of sGlut-PSCs compared with *Magel2-*KO mice (Fig. 6C, D). The GABAergic activity (amplitude and frequency) was not changed in WT mice after an OT-treatment (Fig. 6E, F). In *Magel2-*KO, OT-treatment decreased (×1.9 less) significantly the frequency of sGABA-PSCs restoring a frequency similar to WT (Fig. 6E); no effect OT-treatment was observed on the amplitude of sGABA-PSCs (Fig. 6F). These results show that OT administration in the first week of life normalized the frequency of spontaneous GABAergic activity in *Magel2-*KO neurons. Neonatal OT-treatment strongly reduced the frequency of glutamatergic activity in *Magel2-*KO and the reduction was even stronger on the amplitude and frequency in WT mice. Notably, WT mice had normal behaviors (Supplementary Fig. 2).

### A delay of the excitatory-to-inhibitory developmental GABA shift in *Magel2-*KO hippocampal neurons is corrected by neonatal OT-administration

**Fig. 7:**
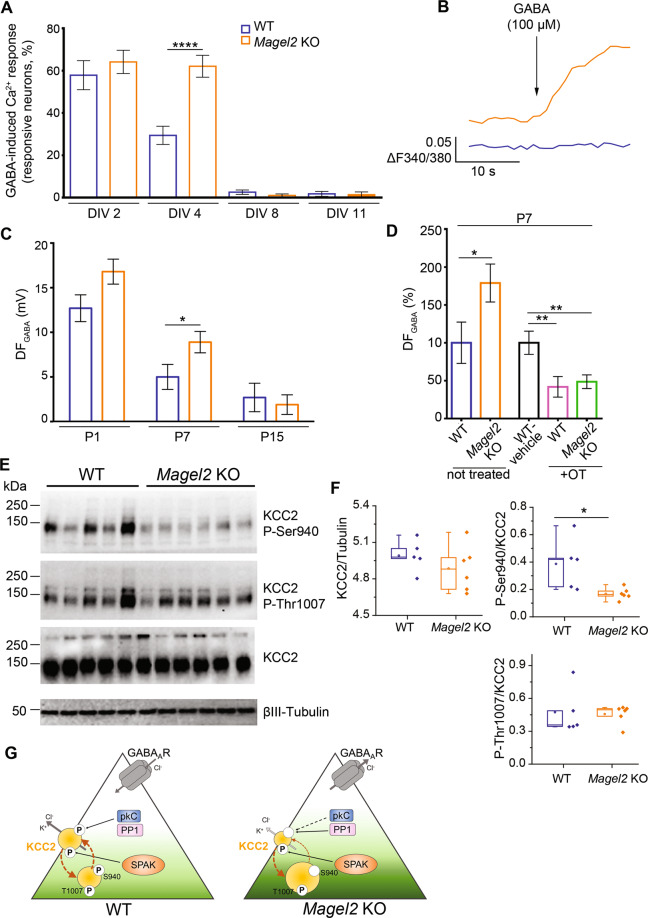
The excitatory-to-inhibitory developmental GABA shift in *Magel2* KO versus WT hippocampi and the effect on an OT-treatment. Abundance and phosphorylation state of KCC2 in WT and Magel2 KO pups in relation with this GABA shift. **A**, **B** GABA-induced Ca^2+^ responses in *Magel2* KO developing hippocampal neuronal cultures versus WT. **A** Percentage of WT and *Magel2* KO E18 hippocampal neurons showing GABA-induced Ca^2+^ responses at selected in vitro days (DIV). **B** Representative traces of [Ca^2+^]_i_ variations (delta F340/380) in DIV4 WT and *Magel2* KO neurons upon 100 μM GABA administration. Data are presented in histograms with mean ± SEM; unpaired *t* test with Welch’s correction: ****P < 0.0001. **C**, **D** Measures of the driving force for GABA (DF_GABA_), using cell-attached recordings of single GABA_A_ channels, of aCA3d pyramidal neurons. **C** Average values of the DF_GABA_ at P1, P7, and P15 in *Magel2* KO (P1 *N* = 3, *n* = 20; P7: *N* = 7, *n* = 56; P15: *N* = 4, *n* = 29) versus WT (P1 *N* = 3, *n* = 19; P7: *N* = 6, *n* = 42; P15: *N* = 3, *n* = 23) mice. Data are presented in histograms with mean ± SEM; unpaired *t* test with Welch’s correction: *P < 0.05. **D** Graph reporting the relative changes of the DF_GABA_ at P7 in untreated *Magel2*-KO mice (*N* = 7, *n* = 56) compared with WT mice (*N* = 6, *n* = 42) and in OT-treated WT (*N* = 3, *n* = 37) and *Magel2*-KO (*N* = 4, *n* = 56) mice compared with WT-vehicle (*N* = 3, *n* = 37) mice. N number of mice and n number of recorded cells. One-way ANOVA + Dunnett’s post hoc test: **P < 0.01. **E**, **F** Abundance and phosphorylation state of KCC2 in WT and *Magel2* KO pups. **E** Immunoblot analysis of WT (*N* = 5) and *Magel2* KO (*N* = 6) hippocampi of P7 mice with pan-KCC2 antibody or phosphorylation site-specific antibodies recognizing P-Ser^940^ or P-Thr^1007^ of KCC2. A neuron-specific ß3 tubulin antibody was used to normalize the quantity of proteins. Numbers on the left indicate molecular weight. **F** Box-plots report band intensities from (**E**) as Q2 (Q1, Q3), with scattered plot showing individual data points. Mann–Whitney test, *P < 0.05. **G** A model of KCC2-dependent control of neuronal Cl^−^ in *Magel2* KO pups. At P7, the surface expression of KCC2, that determines its ion-transport activity, depends on the ratio of reciprocal phosphorylation of its Ser^940^ and Thr^1007^ residues. Ser^940^ phosphorylation increases KCC2’s cell surface stability, whereas Thr^1007^ phosphorylation exerts opposite to Ser^940^ effect and favors internalization (brown arrows). Compared to WT, the CA3 neurons in hippocampi from *Magel2* KO mice are characterized by depolarizing action of GABA (e.g., activation of GABA generates Cl^-^ efflux) that reflects higher [Cl^-^]_i_. In *Magel2* KO hippocampi the amount of KCC2’s Ser^940^ phosphorylation is significantly lower as compared to WT hippocampi whereas the amount of phosphorylated Thr^1007^ remains unchanged. Respectively, the decreased P-Ser^940^/P-Thr^1007^ ratio is predicted to result in predominance of KCC2 internalization over surface expression. We hypothesize, as a consequence of the decreased amount of surface expressed KCC2, the Cl^−^ extrusion is decreased, leading to increased [Cl^−^]_i_ and a depolarizing shift of GABA. The model includes also important components that are known to control the level of Ser^940^ and Thr^1007^ phosphorylation. Ser^940^ is directly phosphorylated by kinase C (pkC) and dephosphorylated under pathology conditions by protein phosphatase type 1 (PP1). Thr^1007^ is directly phosphorylated by SPAK. Statistical analysis is reported in Supplementary Table 6.

Because *Oxtr* and *Magel2* are co-expressed in aCA2/CA3d hippocampus in infancy (P7), and because in *Oxtr* KO mice [36], as in several models [38] of autism, the depolarizing to hyperpolarizing developmental GABA shift is delayed, we investigated the GABA-shift timing in *Magel2-*KO pups. First, we performed calcium (Ca^2+^) imaging experiments by measuring the percentage of neurons showing GABA-induced Ca^2+^ responses in developing hippocampal neuronal cultures of *Magel2-*KO and WT embryos collected at E18.5 d.p.c. (DIV0 for days in vitro 0) (Fig. 7A, B). At DIV4, we found a significant two-fold higher proportion of *Magel2-*KO neurons increasing Ca^2+^ upon GABA stimulation compared with WT. This difference was abolished at DIV8 and DIV11 with a marked decrease of the percentage of responsive neurons in both genotypes. In *Magel2-*KO, KCl-stimulation did not increase Ca^2+^ responses at DIV2, DIV4 and DIV8 compared to WT (Supplementary Fig. 7B), suggesting that the voltage-operated calcium channels were not responsible for the higher proportion of *Magel2-*KO GABA evoked neuronal responses upon stimulation. Altogether, these results showed a developmental delay in GABA-induced Ca^2+^ responses in cultures of *Magel2-*KO embryonic hippocampal neurons and suggest a developmental GABA-shift delay in *Magel2-*KO hippocampal neurons.

Cell-attached recordings of single GABA_A_ channels/receptors were then performed in acute brain slices in order to measure the Driving Force of GABA_A_ (DF_GABA_) and therefore the hyperpolarizing or depolarizing response of GABA under conditions where intracellular chloride levels are not altered by the recording pipette. At P1, we observed a tendency to an increase of DF_GABA_ in *Magel2-*KO compared with WT; at P7, the DF_GABA_ was significantly increased (x1.8) in mutant neurons but this difference was abolished at P15 (Fig. 7C). Since *Magel2* and *Oxtr* are expressed in interneurons (INs), in which a GABA shift has been also described [39], we also measured the DF_GABA_ in INs and observed similar values in CA3 interneurons of mutant and WT mice (Supplementary Fig. 7C). At P7, the resting membrane potential (Supplementary Fig. 7D), the capacitance (Supplementary Fig. 7E) and the conductance (Supplementary Fig. 7F) did not differ statistically between WT and *Magel2*-KO neurons. Altogether these data suggest a transient higher GABA depolarizing activity at P7 in CA3-pyramidal neurons of *Magel2-*KO pups and consequently a delay in GABA shift. Then, at P7, we assessed the effect of the neonatal OT-administration in *Magel2-*KO and WT neonates compared with WT-vehicle pups. Both *Magel2-*KO + OT and WT + OT mice showed a significant decrease (x2.4 less and x2 less, respectively) in the DF_GABA_ values compared with WT-vehicle (Fig. 7D), suggesting a reduction in GABA depolarizing activity following a neonatal OT-administration.

### Post-translational changes in cation-chloride co-transporter KCC2 in *Magel2-*KO hippocampus

We then asked if the altered DF_GABA_ values could be due to alteration in the expression of the neuronal transporters of Cl^−^ in *Magel2-*KO mice. The neuronal level of [Cl^−^]_i_ and Cl-dependent depolarizing or hyperpolarizing strength of GABA are determined by complex mechanism involving primarily Cl^−^ extrusion by potassium/chloride co-transporter type 2 (KCC2) whose expression increases progressively during neuronal maturation [40]. In developing WT hippocampal neurons, the emerging activity of KCC2 contributes to progressive lowering of [Cl^−^]_i_ that at P7 shifts GABA action from depolarizing to hyperpolarizing. As consequence, the activation of GABA_A_R produces neuronal Cl^-^ influx.

Quantitative western blot analysis of the total KCC2 protein expression in hippocampi of P7 mice did not reveal statistically significant difference of the amount of KCC2 between WT and *Magel2-*KO animals (Fig. 7E, F). However, the ion-transport activity of KCC2 and its stability at the cellular plasma membrane depend on post-translational modifications of multiple phosphorylation sites [41]. We therefore used phospho-site-specific antibodies, previously shown to quantitatively monitor changes in KCC2 phosphorylation [42, 43]. Currently, a limited number of such phospho-specific antibody is available. They are directed against the well-known KCC2’s phospho-sites Ser^940^ [44] and Thr^1007^ [42, 43]. Western blot analysis revealed that the *Magel2-*KO hippocampi (as compared to WT) were characterized by significantly decreased amount of the phosphorylated form of Ser^940^ (P-Ser^940^). The amount of phosphorylated Thr^1007^ (P-Thr^1007^) was not statistically different, although a small but not significant increase was observed in *Magel2-*KO mice (Fig. 7E, F). At P7, the decreased P-Ser^940^/P-Thr^1007^ ratio in *Magel2-*KO mice may thus result in predominance of KCC2 internalization over surface expression. As a consequence of the decreased amount of surface expressed molecules, the Cl^-^ extrusion ability of KCC2 is decreased, causing an increase of [Cl^−^]_i_ and could induce a depolarizing shift of GABA described above (Fig. 7G).

## Discussion

Here we investigated, in hippocampus, the mechanisms by which peripheral administration of OT in neonates acts to restore normal social memory in *Magel2-*KO mice. Peripheral OT-administration in neonates permanently rescued almost all the hippocampal alterations (quantity of OT-binding sites, number of SST-positive interneurons and an increase in the GABAergic activity of pyramidal neurons) that we have characterized and that are associated with the increase of GABAergic activity observed in *Magel2-*KO adult mice; but a decrease of glutamatergic activity is still present. Those alterations are related with the OT-signaling pathway and relevant to explain the loss of social memory. However, a significant impact of OT-treatment, reducing the glutamatergic activity, was also observed in wild-type mice but all performed behavioral tests were normal. A significant effect of OT-administration on the delayed excitatory-to-inhibitory developmental GABA shift (at P7), delays that are observed in various neurodevelopmental disorders, underlies the therapeutic use of neonatal oxytocin in these disorders.

### The effect of peripheral OT-administration in *Magel2-*KO neonates

Adding to our previous work on OT-treatment in *Magel2*-KO neonates, we have shown long-term and beneficial effects of a peripheral administration of OT in *Magel2-*KO neonates that rescues nearly all social and cognition deficits described in adult *Magel2-*KO mice [16], including social memory described here. However, previously, we did not investigate neither the neurobiological causes of these deficits nor the effects of OT on those alterations. We focused this study on social memory because the mechanisms by which OT controls social memory via the OXTR-expressing neurons in hippocampus are known. We clearly demonstrated that: (1) the neurobiological alterations found in *Magel2-*KO mice involve the hippocampal brain OT-circuitry and (2) peripheral administration of OT in neonates impacts this circuitry. Thus, those results give a clear and positive response to the debated question on the action of peripheral administration of OT on the brain. Does peripheral OT produce direct central effects by crossing the Blood–Brain–Barrier (BBB) or indirect effects via a peripheral route? It might be both. Recent studies suggest that peripheral OT goes through the BBB via an active mechanism using RAGE transporters [45, 46] and/or a passive mechanism, in neonates, when this barrier is more permeable, and/or perhaps by modulating peripheral sensory afferents [47–49] to enhance endogenous production and/or release of OT.

The observed long-lasting OT effects could result from a strong impact of OT administration in key developmental hippocampal processes such as the developmental GABA shift (as discussed below) and can also be achieved by epigenetic modifications that impact gene expression such as *Oxtr* expression, as observed in prairie voles following maternal OT administration [50]. Transcriptomic and proteomic studies at different developmental ages would help to understand the life-long effect of an early OT-treatment in mutant and WT mice.

### The lack of *Magel2* alters the OT-system: causes and consequences

*Magel2* is expressed in hypothalamic OT neurons and, in *Magel2*-KO neonates, we observed a deficit in the quantity of the mature form of OT although we detected an accumulation of the intermediate non-mature forms of OT suggesting a problem of hormonal processing [18]. Here we showed a co-expression of *Magel2* and *Oxtr* transcripts in the hippocampal neurons. Thus, given the role of *MAGEL2* in ubiquitination, actin regulation and endosomal sorting processes [51], the absence of *Magel2* expression could induce post-translational modifications of various processes in OT and OXTR-expressing neurons, suggesting that dysregulation of the OT-system in *Magel2-*KO mice goes beyond OT expression.

Here we showed that the excitatory-to-inhibitory GABAergic developmental sequence is transiently delayed in *Magel2*-KO pyramidal neurons during the first week of life, with GABA_A_-mediated responses more depolarizing in *Magel2-*KO than WT mice at P7. We further showed that this electrophysiological deficit corresponds well with decreased functional KCC2 at the cell membrane, indicated by a deficit of KCC2 phosphorylation (on Ser^940^). Notably, Ser^940^-phosphorylation is controlled by OXTR activation via a PKC-dependent pathway and allows translocation of KCC2 to the cell membrane [36], enhancing KCC2 mediated Cl^−^ transport [44, 52]. This mechanism is relevant to control the GABAergic developmental sequence in vitro and possibly in vivo [53].

The functional consequences of this delayed developmental GABA shift in *Magel2-*KO pyramidal neurons are not clearly established. However, there are compelling reasons to suspect that transient disruption of GABAergic maturation in the immediate postnatal period might be sufficient to permanently alter neural circuit dynamics. Indeed, P7 is a critical milestone in the development of GABAergic neurons in the mouse neocortex and hippocampus, characterized by major changes in network dynamics (e.g., the end of in vitro giant-depolarizing-potentials [54] and in vivo early sharp waves [55]), intrinsic membrane properties (e.g., input resistance) and synaptic connectivity [56]. Altogether those data suggest that the absence of *Magel2* delays neuronal maturation during this critically vulnerable period of brain development, resulting in a distinct adult phenotype. Whether the delay of the GABA shift alone is sufficient to derail neurotypical developmental trajectory remains a key question for future study: notably, similar or longer GABA-shift delays have been observed in several models of autism [57–59] and in *Oxtr* KO mouse models [36]. Recently, Kang et al. [60] showed, in a *Disc1* KO mouse model, that elevated depolarizing GABA signaling is a precursor for the later E/I imbalance (in favor of inhibition) and social impairment. Similarly, we previously showed that, in a KCC2 mutant mouse, the GABA-shift delay is responsible for the E/I alteration [53].

Importantly, OT-treatment increases the GABA hyperpolarizing activity at P7, during the period of the excitatory-to-inhibitory GABA shift and might accelerate this shift. This effect of OT-treatment might modify the maturation of the hippocampal circuitry.

### The E/I ratio and social behavior

Reductions in synaptic signal-to-noise ratio in cortical and hippocampal pyramidal neurons, driven by a change in the ratio of dendritic excitatory and inhibitory synapses, are widely thought to contribute to reduced efficiency of signal processing in ASD, a mechanism known as the E/I ratio hypothesis [61]. We confirm E/I imbalance characterized by increased GABAergic activity and lower glutamatergic activity in CA3 neurons in *Magel2-*KO mice, consistent with observations made in some ASD models [62–64]. Furthermore, we report that perinatal OT administration restored normal GABAergic activity in *Magel2-*KO mice without improving glutamatergic transmission. Unexpectedly, perinatal OTtreatment has a significant impact on the WT neurons inducing a strong reduction of glutamatergic activity without affecting GABAergic activity. This is a significant observation, because it shows that, although the ASD-like behavior of *Magel2-*KO animals is correlated with a change in E/I ratio, E/I imbalance in OT-treated WT animals was not sufficient to drive detectable changes in social behavior or in the novel object recognition test. We therefore propose that E/I imbalance characterized by isolated decreased spontaneous glutamatergic transmission is unlikely to underlie the ASD traits investigated here, and suggest that an upper threshold of GABAergic or glutamatergic activity, but not the E/I ratio per se, may be important for normal development.

### Role of oxytocin receptors and somatostatin neurons

In adult *Magel2-*KO mice we observed increased OT-binding in the DG and CA2/CA3 regions of the anterior hippocampus compared to WT mice. OT administration in *Magel2-*KO neonates normalized hippocampal OT-binding sites in adulthood, suggesting that the increased expression of OXTR observed in *Magel2-*KO hippocampus may be a consequence of the reduced OT production reported in these animals [18]. This observation supports the idea that life-long OXTR expression is to some extent determined by early life OT-binding, described as a “hormonal imprinting” effect [50, 65].

Since DG and CA2/CA3 hippocampal OXTRs are expressed in PV and SST interneurons, we quantified those populations and found a significant increase in the number of aDG and aCA2/CA3d SST+ neurons in mutant mice, while the number of PV+ interneurons was not modified. OT-treatment of pups normalized the number of SST-expressing neurons in *Magel2-*KO adults, revealing a causal link between the administration of OT in infancy and the quantity of SST+ neurons in adulthood. This result may reflect actual changes in the number of SST+ neurons, or alternatively changes in SST expression and hence more reliable detection of SST-synthesizing neurons. Interestingly, OT modulates the activity of the SST+ neurons, increasing the excitability of SST interneurons [66] but no studies report an effect of OT on SST production.

SST interneurons have recently been shown to play a role in the modulation of social behavior [67, 68] and a link between altered social memory and an increase in SST cell number has been recently suggested in LPS-treated female neonates [69]. It is tempting to speculate that OXTR-transmission regulates the activity of SST hippocampal interneurons and the production/release of mature SST and impacts social memory. Further work is needed to fully characterize the role of OXTRs on SST interneurons in relation with social memory.

### The lack of short-term social memory in wild-type females

In wild-type females, the results on social exploration and discrimination are in agreement with previous studies [37, 70]. The lack of preference by wild-type females for a novel female mouse in the short-term social memory task is puzzling. This result was obtained in four different cohorts (two are not shown and the OT-treated cohort is excluded), indicating that it is highly reproducible. We cannot compare our results with other studies due to the lack of similar studies reported in the literature using this three-chamber paradigm with the short-term social memory task (Raam et al. [25] studied only males). However, our present results clearly question the validity of this test for assessing social memory in females. In addition, it suggests a new important question that will need to be addressed: do adult females prefer to interact with familiars and are not interested in exploring novel same-sex congeners? Indeed, in the discrimination test, females showed lower levels of novelty exploration compared with males, as observed in this study and by Karlsson et al. [37]. Interestingly, Moy et al. [71] did not find this sex difference when they tested juvenile females and used juvenile males as stimuli, suggesting that it may be an age-related behavior. We should consider that social memory is stimulus-dependent and sexual dimorphism exists in social behavior to interact with a novel conspecific of the same sex. A role for oxytocin in short-term social memory has been clearly revealed in males but females have not been properly studied. Interestingly, it has also recently been shown that the action of OT on the same neurons regulates distinct behaviors by sex [72, 73]. Further studies are needed to clarify why females show low interactions towards novel conspecifics and what are the differences in social memory between male and female mice during the different phases of life.

## Conclusions

Oxytocin deficiency, present in the *Magel2-*KO mouse model and in PWS, has also been frequently described in rodent models of ASD [2]. Recently, evidence for a unifying role of oxytocin in pathogenic mechanisms responsible for social impairments across a broad range of autism etiologies have been provided [74, 75]. Thus, our results demonstrate that peripheral OT-administration in a critical period of time, after birth, represents a viable therapeutic strategy for patients with SYS or PWS and possibly other neurodevelopmental disorders.

## Supplementary information

Supplemental Information - Materials and Methods

Supplemental Information - Statistical Tables

Supplementary Figure 1

Supplementary Figure 2

Supplementary Figure 3

Supplementary Figure 4

Supplementary Figure 5

Supplementary Figure 6

Supplementary Figure 7

## References

[1] Carter CS, Kenkel WM, MacLean EL, Wilson SR, Perkeybile AM, Yee JR (2020). Is oxytocin “nature’s medicine”?. Pharmacol Rev.

[2] Wagner S, Harony-Nicolas H (2018). Oxytocin and animal models for autism spectrum disorder. Curr Top Behav Neurosci.

[3] Muscatelli F, Desarmenien MG, Matarazzo V, Grinevich V (2018). Oxytocin signaling in the early life of mammals: link to neurodevelopmental disorders associated with ASD. Curr Top Behav Neurosci.

[4] Winslow JT, Hearn EF, Ferguson J, Young LJ, Matzuk MM, Insel TR (2000). Infant vocalization, adult aggression, and fear behavior of an oxytocin null mutant mouse. Horm Behav.

[5] Ferguson JN, Aldag JM, Insel TR, Young LJ (2001). Oxytocin in the medial amygdala is essential for social recognition in the mouse. J Neurosci.

[6] Takayanagi Y, Yoshida M, Bielsky IF, Ross HE, Kawamata M, Onaka T (2005). Pervasive social deficits, but normal parturition, in oxytocin receptor-deficient mice. Proc Natl Acad Sci USA.

[7] Sala M, Braida D, Lentini D, Busnelli M, Bulgheroni E, Capurro V (2011). Pharmacologic rescue of impaired cognitive flexibility, social deficits, increased aggression, and seizure susceptibility in oxytocin receptor null mice: a neurobehavioral model of autism. Biol Psychiatry.

[8] Sala M, Braida D, Donzelli A, Martucci R, Busnelli M, Bulgheroni E (2013). Mice heterozygous for the oxytocin receptor gene (Oxtr(+/-)) show impaired social behaviour but not increased aggression or cognitive inflexibility: evidence of a selective haploinsufficiency gene effect. J Neuroendocrinol.

[9] Jin D, Liu HX, Hirai H, Torashima T, Nagai T, Lopatina O (2007). CD38 is critical for social behaviour by regulating oxytocin secretion. Nature.

[10] Liu HX, Lopatina O, Higashida C, Tsuji T, Kato I, Takasawa S (2008). Locomotor activity, ultrasonic vocalization and oxytocin levels in infant CD38 knockout mice. Neurosci Lett.

[11] Boccaccio I, Glatt-Deeley H, Watrin F, Roeckel N, Lalande M, Muscatelli F (1999). The human MAGEL2 gene and its mouse homologue are paternally expressed and mapped to the Prader-Willi region. Hum Mol Genet.

[12] Schaaf CP, Gonzalez-Garay ML, Xia F, Potocki L, Gripp KW, Zhang B, et al. Truncating mutations of MAGEL2 cause Prader-Willi phenotypes and autism. Nat Genet. 2013;45:1405–8. 10.1038/ng.2776 PMC3819162 24076603

[13] McCarthy J, Lupo PJ, Kovar E, Rech M, Bostwick B, Scott D, et al. Schaaf-Yang syndrome overview: report of 78 individuals. Am J Med Genet A. 2018;176:2564–74. 10.1002/ajmg.a.40650 PMC6585857 30302899

[14] Thomason MM, McCarthy J, Goin-Kochel RP, Dowell LR, Schaaf CP, Berry LN (2020). Neurocognitive and neurobehavioral phenotype of youth with Schaaf-Yang syndrome. J Autism Dev Disord.

[15] Fountain MD, Schaaf CP. Prader-Willi syndrome and Schaaf-Yang syndrome: neurodevelopmental diseases intersecting at the MAGEL2 gene. Diseases. 2016;4:2. 10.3390/diseases4010002 PMC5456300 28933382

[16] Meziane H, Schaller F, Bauer S, Villard C, Matarazzo V, Riet F (2015). An early postnatal oxytocin treatment prevents social and learning deficits in adult mice deficient for Magel2, a gene involved in prader-willi syndrome and autism. Biol Psychiatry.

[17] Fountain MD, Aten E, Cho MT, Juusola J, Walkiewicz MA, Ray JW (2017). The phenotypic spectrum of Schaaf-Yang syndrome: 18 new affected individuals from 14 families. Genet Med.

[18] Schaller F, Watrin F, Sturny R, Massacrier A, Szepetowski P, Muscatelli F (2010). A single postnatal injection of oxytocin rescues the lethal feeding behaviour in mouse newborns deficient for the imprinted Magel2 gene. Hum Mol Genet.

[19] Dai YC, Zhang HF, Schon M, Bockers TM, Han SP, Han JS (2018). Neonatal oxytocin treatment ameliorates autistic-like behaviors and oxytocin deficiency in valproic acid-induced rat model of autism. Front Cell Neurosci.

[20] Penagarikano O, Lazaro MT, Lu XH, Gordon A, Dong H, Lam HA (2015). Exogenous and evoked oxytocin restores social behavior in the Cntnap2 mouse model of autism. Sci Transl Med.

[21] Francis SM, Sagar A, Levin-Decanini T, Liu W, Carter CS, Jacob S. Oxytocin and vasopressin systems in genetic syndromes and neurodevelopmental disorders. Brain Res. 2014;1580:199–218. 10.1016/j.brainres.2014.01.021 PMC4305432 24462936

[22] Mansouri M, Pouretemad H, Roghani M, Wegener G, Ardalan M. Autistic-like behaviours and associated brain structural plasticity are modulated by oxytocin in maternally separated rats. Behav Brain Res. 2020;393:112756. 10.1016/j.bbr.2020.112756 32535183

[23] Johnson ZV, Walum H, Xiao Y, Riefkohl PC, Young LJ (2017). Oxytocin receptors modulate a social salience neural network in male prairie voles. Horm Behav.

[24] Caldwell HK, Aulino EA, Freeman AR, Miller TV, Witchey SK (2017). Oxytocin and behavior: lessons from knockout mice. Dev Neurobiol.

[25] Raam T, McAvoy KM, Besnard A, Veenema AH, Sahay A (2017). Hippocampal oxytocin receptors are necessary for discrimination of social stimuli. Nat Commun.

[26] Lin YT, Hsieh TY, Tsai TC, Chen CC, Huang CC, Hsu KS (2018). Conditional deletion of hippocampal CA2/CA3a oxytocin receptors impairs the persistence of long-term social recognition memory in mice. J Neurosci.

[27] Okuyama T (2018). Social memory engram in the hippocampus. Neurosci Res.

[28] Cilz NI, Cymerblit-Sabba A, Young WS. Oxytocin and vasopressin in the rodent hippocampus. Genes Brain Behav. 2018;18:e12535. 10.1111/gbb.12535 30378258

[29] Young WS, Song J (2020). Characterization of oxytocin receptor expression within various neuronal populations of the mouse dorsal hippocampus. Front Mol Neurosci.

[30] Tirko NN, Eyring KW, Carcea I, Mitre M, Chao MV, Froemke RC (2018). Oxytocin transforms firing mode of CA2. Neuron.

[31] Vaidyanathan R, Hammock EA (2017). Oxytocin receptor dynamics in the brain across development and species. Dev Neurobiol.

[32] Newmaster KT, Nolan ZT, Chon U, Vanselow DJ, Weit AR, Tabbaa M (2020). Quantitative cellular-resolution map of the oxytocin receptor in postnatally developing mouse brains. Nat Commun.

[33] Ripamonti S, Ambrozkiewicz MC, Guzzi F, Gravati M, Biella G, Bormuth I, et al. Transient oxytocin signaling primes the development and function of excitatory hippocampal neurons. eLife. 2017;6:e22466. 10.7554/eLife.22466 PMC5323041 28231043

[34] Rice LJ, Einfeld SL, Hu N, Carter CS (2018). A review of clinical trials of oxytocin in Prader-Willi syndrome. Curr Opin Psychiatry.

[35] Tauber M, Boulanouar K, Diene G, Cabal-Berthoumieu S, Ehlinger V, Fichaux-Bourin P, et al. The use of oxytocin to improve feeding and social skills in infants with Prader-Willi syndrome*.* Pediatrics. 2017;139:e20162976. 10.1542/peds.2016-2976 28100688

[36] Leonzino M, Busnelli M, Antonucci F, Verderio C, Mazzanti M, Chini B (2016). The timing of the excitatory-to-inhibitory GABA switch is regulated by the oxytocin receptor via KCC2. Cell Rep.

[37] Karlsson SA, Studer E, Kettunen P, Westberg L (2016). Neural androgen receptors modulate gene expression and social recognition but not social investigation. Front Behav Neurosci.

[38] Ben-Ari Y (2015). Is birth a critical period in the pathogenesis of autism spectrum disorders?. Nat Rev Neurosci.

[39] Tyzio R, Minlebaev M, Rheims S, Ivanov A, Jorquera I, Holmes GL (2008). Postnatal changes in somatic gamma-aminobutyric acid signalling in the rat hippocampus. Eur J Neurosci.

[40] Rivera C, Voipio J, Payne JA, Ruusuvuori E, Lahtinen H, Lamsa K (1999). The K+/Cl- co-transporter KCC2 renders GABA hyperpolarizing during neuronal maturation. Nature.

[41] Zhang J, Cordshagen A, Medina I, Nothwang HG, Wisniewski JR, Winklhofer M (2020). Staurosporine and NEM mainly impair WNK-SPAK/OSR1 mediated phosphorylation of KCC2 and NKCC1. PLoS ONE.

[42] Friedel P, Kahle KT, Zhang J, Hertz N, Pisella LI, Buhler E (2015). WNK1-regulated inhibitory phosphorylation of the KCC2 cotransporter maintains the depolarizing action of GABA in immature neurons. Sci Signal.

[43] de Los Heros P, Alessi DR, Gourlay R, Campbell DG, Deak M, Macartney TJ (2014). The WNK-regulated SPAK/OSR1 kinases directly phosphorylate and inhibit the K+-Cl- co-transporters. Biochem J.

[44] Lee HH, Deeb TZ, Walker JA, Davies PA, Moss SJ (2011). NMDA receptor activity downregulates KCC2 resulting in depolarizing GABAA receptor-mediated currents. Nat Neurosci.

[45] Yamamoto Y, Liang M, Munesue S, Deguchi K, Harashima A, Furuhara K (2019). Vascular RAGE transports oxytocin into the brain to elicit its maternal bonding behaviour in mice. Commun Biol.

[46] Yamamoto Y, Higashida H (2020). RAGE regulates oxytocin transport into the brain. Commun Biol.

[47] Vaidyanathan R, Hammock EAD (2020). Oxytocin receptor gene loss influences expression of the oxytocin gene in C57BL/6J mice in a sex- and age-dependent manner. J Neuroendocrinol.

[48] Vaidyanathan R, Schaller F, Muscatelli F, Hammock EAD (2020). Colocalization of Oxtr with Prader-Willi syndrome transcripts in the trigeminal ganglion of neonatal mice. Hum Mol Genet.

[49] Tabbaa M, Hammock EAD (2020). Orally administered oxytocin alters brain activation and behaviors of pre-weaning mice. Horm Behav.

[50] Kenkel WM, Perkeybile AM, Yee JR, Pournajafi-Nazarloo H, Lillard TS, Ferguson EF (2019). Behavioral and epigenetic consequences of oxytocin treatment at birth. Sci Adv.

[51] Tacer KF, Potts PR (2017). Cellular and disease functions of the Prader-Willi Syndrome gene MAGEL2. Biochem J.

[52] Kahle KT, Deeb TZ, Puskarjov M, Silayeva L, Liang B, Kaila K (2013). Modulation of neuronal activity by phosphorylation of the K-Cl cotransporter KCC2. Trends Neurosci.

[53] Pisella LI, Gaiarsa JL, Diabira D, Zhang J, Khalilov I, Duan J, et al. Impaired regulation of KCC2 phosphorylation leads to neuronal network dysfunction and neurodevelopmental pathology. Sci Signal. 2019;12:eaay0300. 10.1126/scisignal.aay0300 PMC7192243 31615899

[54] Ben-Ari Y, Spitzer NC (2004). Nature and nurture in brain development. Trends Neurosci.

[55] Leinekugel X, Khazipov R, Cannon R, Hirase H, Ben-Ari Y, Buzsaki G (2002). Correlated bursts of activity in the neonatal hippocampus in vivo. Science.

[56] Shi Y, Grieco SF, Holmes TC, Xu X. Development of local circuit connections to hilar mossy cells in the mouse dentate gyrus. eNeuro. 2019;6:ENEURO.0370-18.2019. 10.1523/ENEURO.0370-18.2019 PMC6439204 30937358

[57] He Q, Nomura T, Xu J, Contractor A (2014). The developmental switch in GABA polarity is delayed in fragile X mice. J Neurosci.

[58] Ben-Ari Y (2014). The GABA excitatory/inhibitory developmental sequence: a personal journey. Neuroscience.

[59] Banerjee A, Rikhye RV, Breton-Provencher V, Tang X, Li C, Li K, et al. Jointly reduced inhibition and excitation underlies circuit-wide changes in cortical processing in Rett syndrome. Proc Natl Acad Sci USA. 2016;113:E7287–96. 10.1073/pnas.1615330113 PMC5135376 27803317

[60] Kang E, Song J, Lin Y, Park J, Lee JH, Hussani Q (2019). Interplay between a mental disorder risk gene and developmental polarity switch of GABA action leads to excitation-inhibition imbalance. Cell Rep.

[61] Sohal VS, Rubenstein JLR (2019). Excitation-inhibition balance as a framework for investigating mechanisms in neuropsychiatric disorders. Mol Psychiatry.

[62] Harrington AJ, Raissi A, Rajkovich K, Berto S, Kumar J, Molinaro G, et al. MEF2C regulates cortical inhibitory and excitatory synapses and behaviors relevant to neurodevelopmental disorders. eLife. 2016;5:e20059. 10.7554/eLife.20059 PMC5094851 27779093

[63] Tabuchi K, Blundell J, Etherton MR, Hammer RE, Liu X, Powell CM (2007). A neuroligin-3 mutation implicated in autism increases inhibitory synaptic transmission in mice. Science.

[64] Unichenko P, Yang JW, Kirischuk S, Kolbaev S, Kilb W, Hammer M, et al. Autism related neuroligin-4 knockout impairs intracortical processing but not sensory inputs in mouse barrel cortex. Cerebral Cortex. 2018;28:2873–86. 10.1093/cercor/bhx165 29106499

[65] Carter CS (2003). Developmental consequences of oxytocin. Physiol Behav.

[66] Maldonado PP, Nuno-Perez A, Kirchner JH, Hammock E, Gjorgjieva J, Lohmann C (2021). Oxytocin shapes spontaneous activity patterns in the developing visual cortex by activating somatostatin interneurons. Curr Biol.

[67] Perez SM, Boley A, Lodge DJ (2019). Region specific knockdown of parvalbumin or somatostatin produces neuronal and behavioral deficits consistent with those observed in schizophrenia. Transl psychiatry.

[68] Scheggia D, Manago F, Maltese F, Bruni S, Nigro M, Dautan D (2020). Somatostatin interneurons in the prefrontal cortex control affective state discrimination in mice. Nat Neurosci.

[69] Smith C, Kingsbury M, Dziabis J, Hanamsagar R, Malacon K, Tran J, et al. Neonatal immune challenge induces female-specific changes in social behavior and somatostatin cell number. Brain Behav Immun. 2020;90:332–45. 10.1016/j.bbi.2020.08.013 PMC7556772 32860938

[70] Chari T, Griswold S, Andrews NA, Fagiolini M (2020). The stage of the estrus cycle is critical for interpretation of female mouse social interaction behavior. Front Behav Neurosci.

[71] Moy SS, Nadler JJ, Perez A, Barbaro RP, Johns JM, Magnuson TR (2004). Sociability and preference for social novelty in five inbred strains: an approach to assess autistic-like behavior in mice. Genes Brain Behav.

[72] Nakajima M, Gorlich A, Heintz N (2014). Oxytocin modulates female sociosexual behavior through a specific class of prefrontal cortical interneurons. Cell.

[73] Li K, Nakajima M, Ibanez-Tallon I, Heintz N (2016). A cortical circuit for sexually dimorphic oxytocin-dependent anxiety behaviors. Cell.

[74] Hornberg H, Perez-Garci E, Schreiner D, Hatstatt-Burkle L, Magara F, Baudouin S (2020). Rescue of oxytocin response and social behaviour in a mouse model of autism. Nature.

[75] Lewis EM, Stein-O’Brien GL, Patino AV, Nardou R, Grossman CD, Brown M (2020). Parallel social information processing circuits are differentially impacted in autism. Neuron.

