# Supplementary material for: Oxytocin administration in neonates shapes hippocampal circuitry and restores social behavior in a mouse model of autism
Source: Mol Psychiatry. 2021 Jul 21;26(12):7582–95. doi: 10.1038/s41380-021-01227-6 (PMC8872977; doi:10.1038/s41380-021-01227-6)
Supplement: Supplementary file 1 — Supplemental Information - Materials and Methods [file 41380_2021_1227_MOESM1_ESM.docx]

**SUPPLEMENTAL INFORMATION**

**MATERIAL AND METHODS**

**Animals**

*Magel2* is an imprinted gene, with a monoallelic paternal expression. However, to overcome the phenotypic heterogeneity of the heterozygous +m/-p *Magel2 ^tm1.1Mu^*^s^ mouse, due to the stochastic expression of the maternal *Magel2* allele when the paternal allele is deleted ^1^, *Magel2 ^tm1.1Mus^* homozygous (-/-) mice were used. We made this choice to obtain a greater homogeneity in the values, allowing a better analysis of the effects of the mutation.

*Magel2 ^tm1.1Mus^*+/+ (WT) and *Magel2 ^tm1.1Mus^*-/- (*Magel2*-KO) mice were housed in standard conditions, with *ad-libitum* access to food and water. Mice were handled and cared for in accordance with the Guide for the Care and Use of Laboratory Animals (N.R.C., 1996) and the European Communities Council Directive of September 22th 2010 (2010/63/EU, 74). All efforts were made to minimize the number of animals used. *Magel2*-deficient mice were generated as previously described ^2^. Due to the parental imprinting of *Magel2* (paternally expressed only), to obtain heterozygote mice (+m/-p), males carrying the mutation on the maternal allele (-m/+p) were crossed with wild-type C57BL/6J females. To obtain homozygote mice, *Magel2*-KO homozygote males and females were crossed. Importantly, we checked that *Magel2*-KO mothers had a similar maternal behavior as WT mothers. Mice were separated from their mother at P21 and 3-5 mice of same sex and same age were housed in a same cage. All mice were genotyped by PCR starting from DNA extracted from tail snips (around 3 mm), using the following couples of primers: Ml2KO F (5’-CCCTGGGTTGACTGACTCAT-3’) and Ml2KO R (5’-TCTTCTTCCTGGTGGCTTTG-3’) to discriminate the mutant allele from the WT, 71456 F (5'-CACTCGATCACGTATGGCTCCATCA-3') and 71457 R (5'-GATGGCAGGCACTGACTTACATGCTG-3') to discriminate the heterozygous from the homozygous mice.

For testing female mice in the three-chamber test, we considered the observation reported by Meziane ^3^ et al. (2007): spontaneously cycling females revealed that mice housed in the same room in groups cycled simultaneously with the exception of one or two mice per cage. Indeed, in the context of another project, we confirmed this observation in our animals. So, in order to avoid performing the three-chamber test in sexually-receptive mice, before the test we identified the estrous cycle in 2 females (not further tested, using Grunewald–Giemsa preparations of vaginal smear) among our animals; if these sentinels were in non-estrus state, we performed the experiments on the naïve females.

No randomization was applied. Experimenters were blinded to the group allocation during neuroanatomical and electrophysiological experiments and when assessing the outcome for behavioral experiment.

**Behavior**

All the behavioral tests were performed by Phenotype Expertise, Inc. (France) with a behaviorist. The number of tested animals was based on previous publications and Phenotype Expertise experience. EPM, NOR and open field tests were performed on adult mice (10-12 weeks old). For all tests, animals were first acclimated to the behavioral room for 30 minutes.

***Elevated-Plus Maze.*** The EPM is used to assess anxiety state of animals. The device consists of a labyrinth of 4 arms 5 cm wide located 80 cm above the ground. Two opposite arms are open (without wall) while the other two arms are closed by side walls. The light intensity was adjusted to 20 Lux on the open arms. Mice were initially placed on the central platform and left free to explore the cross-shaped labyrinth for 5 minutes. The maze was cleaned and wiped with H_2_O and with 70% ethanol between each mouse. Animal movement was video-tracked using Ethovision software 11.5 (Noldus). Time spent in open and closed arms, the number of entries in open arms, as well as the distance covered, are directly measured by the software.

***Open-field.*** Open-field test was performed in a 40 x 40 cm square arena with an indirect illumination of 60 lux. Mouse movement was video-tracked using Ethovision software 11.5 (Noldus) for 10 minutes. Total distance traveled and time in center (exclusion of a 5 cm border arena) are directly measured by the software. Grooming (time and events) and rearing were manually counted in live using manual functions of the software, by an experimented behaviorist. The open-field arena was cleaned and wiped with H_2_O and with 70% ethanol between each mouse.

***New object recognition.*** The arena used for the novel object recognition test was the same used for the open-field test. The arena was cleaned and wiped with 70% ethanol between each mouse. Two identical objects (50 ml orange corning tube) were placed in the opposite corners of the arena, 10 cm from the side walls. The tested mouse was placed at the opposite side of the arena and allowed to explore the arena for 10 min. After 1h, one object was randomly replaced with another novel object, which was of similar size but differ in the shape and color with the previous object (white and blue lego bricks). Then, the same mouse was placed in the arena and allowed to explore the two objects (a new and an "old" familiar object) for 10 min. The movement of the mice was video-tracked with Ethovision 11.5 software. Time of exploration of both objects (nose located in a 2 cm area around object) was automatically measured by the software.

***Three-chamber social preference test.*** The test was performed as described previously ^4^. The three-chamber apparatus consisted of a Plexiglas box (50x25 cm) with removable floor and partitions dividing the box into three chambers with 5-cm openings between chambers. The task was carried out in four trials. The three-chambers apparatus was cleaned and wiped with 70% ethanol between each trial and each three-chamber test experiments.

In the first trial (habituation), a test mouse was placed in the center of the three-chamber unit, where two empty wire cages were placed in the left and right chambers to habituate the test mouse to arena. The mouse was allowed to freely explore each chamber. The mouse was video-tracked for 5 min using Ethovision software. At the end of the trial, the animal was gently directed to the central chamber with doors closed. In the second trial (social exploration), an 8 weeks old C57BL/6J congener mouse (S1) was placed randomly in one of the two wire cages to avoid a place preference. The second wire cage remained empty (E). Then, doors between chambers were opened and the test mouse was allowed to freely explore the arena for 10 min. At the end of the trial, the animal was gently directed to the central chamber with doors closed. A second 8 weeks old C57BL/6J congener mouse (S2) was placed in the second wire cage for the third trial (social discrimination). Thus, the tested mouse had the choice between a familiar mouse (S1) and a new stranger mouse (S2) for 10 min. At the end of the trial, the mouse was returned to home-cage for 30 min. For the fourth trial (short-term social memory), S2 was replaced by a new stranger mouse (S3), the familiar mouse (S1) stayed the same. The tested mouse was allowed to freely explore the arena for 10 min. Time spent in each chamber and time of contact with each wire cage (with a mouse or empty wire cage) were calculated using Ethovision software. The measure of the real social contact is represented by the time spent in nose-to-nose interactions with the unfamiliar or familiar mouse. This test was performed using grouped-house mice of 4 months old.

**Primary hippocampal cultures**

Embryonic day 18 dissociated hippocampal neurons were obtained from wild-type and *Magel2*-KO timed pregnant mice as previously described ^5^ with slight modifications here described. Briefly, the hippocampi of E18 embryos were dissociated by an enzymatic treatment (0.25% trypsin for 18 min at 37°C) followed by mechanic dissociation with a fire-smoothed Pasteur pipette or p1000µl then p200µl tips. For calcium imaging experiments, 200 000 cell/well (in MW 6 wells) were plated on round 26 mm glass coverslips pre-coated with poly-L-lysine containing Neurobasal medium (Life Technologies) augmented with B27 supplement (2% v/v; Life Technologies), L-glutamine (2mM), penicillin/streptomycin (100U/ml) and 25µM Glutamate. This media was replaced with glutamate-free media after 5 hours. Neurons were then maintained at 37°C in humidified atmosphere (95% air and 5% CO_2_), and half of the medium was refreshed twice a week.

**Calcium imaging recordings**

For calcium imaging experiments, hippocampal neurons were loaded with the membrane-permeable fluorescent Ca^2+^ indicator Fura-2/AM (1 μM; Sigma Aldrich) for 40 min at 37°C, 5% CO_2_. The cells were then placed into the recording chamber of an inverted microscope (Axiovert 100, Zeiss), washed with the extracellular recording solution, KRH buffer, and imaged through a 40x objective (Zeiss). Fura-2/AM was excited at 380 nm and at 340 nm through a Polychrom V, (TILL Photonics GmbH) controlled by the TillVisION software 4.01. Emitted light was acquired at 505nm at 1Hz, and images collected with a CCD Imago-QE camera (TILL Photonics GmbH). The fluorescence ratio F340/380 (ΔF340/380) was used to express Ca^2+^ concentrations in regions of interest (ROI) corresponding to neuronal cell bodies. 100µM GABA was administered in the recording solution and temporal changes in ΔF340/380 were followed. Increases in ΔF340/380 higher than 0.04 units were considered reliable Ca^2+^ responses. After wash with KRH buffer and recover, KCl (50mM) was administered to identify viable neurons. Responses with a ΔF340/380 smaller than 0.1 units were excluded from the analyses. From DIV8 on, 1 μM TTX (Tocris, cat #1069) was added to this extracellular recording solution.

**Hippocampal slice preparation and electrophysiological recordings**

Brains were removed and immersed into ice-cold (2-4°C) artificial cerebrospinal fluid (ACSF) with the following composition (in mM): 126 NaCl, 3.5 KCl, 2 CaCl_2_, 1.3 MgCl_2_, 1.2 NaH_2_PO_4_, 25 NaHCO_3_ and 11 glucose, pH 7.4 equilibrated with 95% O_2_ and 5% CO_2_. Hippocampal slices (400 µm thick) were cut with a vibrating microtome (Leica VT 1000s, Germany) in ice cold oxygenated choline-replaced ACSF and were allowed to recover at least 90 min in ACSF at room (25°C) temperature. Slices were then transferred to a submerged recording chamber perfused with oxygenated (95% O_2_ and 5% CO_2_) ACSF (3 ml/min) at 34°C.

***Whole-cell patch clamp recordings*** were performed from P20-P25 CA3 pyramidal neurons in voltage-clamp mode using an Axopatch 200B (Axon Instrument, USA). To record the spontaneous and miniature synaptic activity, the glass recording electrodes (4-7 MΩ) were filled with a solution containing (in mM): 100 KGluconate, 13 KCl, 10 HEPES, 1.1 EGTA, 0.1 CaCl_2_, 4 MgATP and 0.3 NaGTP. The pH of the intracellular solution was adjusted to 7.2 and the osmolality to 280 mOsmol l^-1^. The access resistance ranged between 15 to 30 MΩ. With this solution, the GABA_A_ receptor-mediated postsynaptic current (GABA-PSCs) reversed at -70mV. GABA-PSCs and glutamate mediated synaptic current (Glut-PSCs) were recorded at a holding potential of -45mV. At this potential GABA-PSC are outwards and Glut-PSCs are inwards. Spontaneous synaptic activity was recorded in control ACSF and miniature synaptic activity was recorded in ACSF supplemented with tetrodotoxin (TTX, 1µM). Spontaneous and miniature GABA-PSCs and Glut-PSCs were recorded with Axoscope software version 8.1 (Axon Instruments) and analyzed offline with Mini Analysis Program version 6.0 (Synaptosoft).

***Single GABA_A_ channel recordings*** were performed at P1, P7 and P15 visually identified hippocampal CA3 pyramidal cells in cell-attached configuration using Axopatch-200A amplifier and pCLAMP acquisition software (Axon Instruments, Union City, CA). Data were low-pass filtered at 2 kHz and acquired at 10 kHz. The glass recording electrodes (4-7 MΩ) were filled with a solution containing (in mM) for recordings of single GABA_A_ channels: NaCl 120, KCl 5, TEA-Cl 20, 4-aminopyridine 5, CaCl_2_ 0.1, MgCl_2_ 10, glucose 10, Hepes-NaOH 10. The pH of pipette solutions was adjusted to 7.2 and the osmolality to 280 mOsmol l^-1^. Analysis of currents trough single channels and current-voltage relationships were performed using Clampfit 9.2 (Axon Instruments) as described ^6^.

**Morphological analysis**

During electrophysiological recordings, biocytin (0.5%, Sigma, USA) was added to the pipette solution for post hoc reconstruction. Images were acquired using a Leica SP5 X confocal microscope, with a 40x objective and 0,5 µm z-step. Neurons were reconstructed three-dimensionally using Neurolucida software version 10 (MBF Bioscience) from 3D stack images. The digital reconstructions were analyzed with the software L-Measure to measure the number of primary branches and the total number of ramifications of each neuron ^7^. Comparisons between groups were done directly in L-Measure.

**Immunohistochemistry and quantification**

WT and mutant mice were deeply anaesthetized with intraperitoneal injection of the ketamine/xylazine mixture and transcardially perfused with 0.9% NaCl saline followed by Antigenfix (Diapath, cat #P0014). Brains were post-fixed in Antigenfix overnight at 4°C and included in agar 4%. 50 μm-thick coronal sections were sliced using a vibratome (Zeiss) and stored in PBS at 4°C. Floating slices (of the hippocampal region corresponding to the coronal level 68 to 78 on Allen Brain Atlas; Bregma-1.355 to 2.355 mm) were incubated for 1 hour with blocking solution containing 0.1% (v/v) Triton X-100, 10% (v/v) normal goat serum (NGS) in PBS, at room temperature. Sections were then incubated with primary antibodies diluted in incubation solution (0.1% (v/v) Triton X-100, 3% (v/v) NGS, in PBS), overnight at 4°C. After 3 x 10 min washes in PBS, brain sections were incubated with secondary antibodies diluted in the incubation solution, for 2 hours at RT. Sections were washed 3 x 10 min in PBS and mounted in Fluoromount-G (EMS, cat #17984-25). Primary antibodies used were: rabbit polyclonal anti-cFos (1:5000, Santa Cruz Biotech, cat #ab190289), goat polyclonal anti-Sst (D20) (1:500, Santa Cruz Biotech, cat #sc-7819), goat polyclonal anti-PV (1:6000, SWANT, cat #PVG213). Fluorochrome-conjugated secondary antibodies used were: goat anti-rabbit Alexa Fluor 647 (1:500, Invitrogen, cat # A32733), goat anti-rabbit Alexa Fluor 488 (1:500, Invitrogen, cat #A-31565), goat anti-mouse Alexa Fluor 488 (1:500, Invitrogen, cat #A21121), donkey anti-goat Alexa Fluor 488 (1:500, Invitrogen, cat # A32814).

For c-Fos, PV and SST quantification, images were acquired using a fluorescence microscope (Zeiss Axioplan 2 microscope with an Apotome module), and z stacks of 8 µm were performed for each section. Counting was performed on the right and left hippocampus for a total of 6-8 sections (cFos) or 10-14 sections (SST) or 4-5 sections (PV) per animal in the hippocampal regions indicated on the figures and corresponding to coronal levels 68 to 78 (Bregma-1.355 to 2.355 mm) on Allen Brain Atlas. Sections were excluded when the tissue was damaged. If more than 20% of sections issued from the same brain resulted damaged, the individual was excluded.

**OT binding assay**

Briefly, slides were pre-incubated for 5 minutes in a solution of 0.2% paraformaldehyde in phosphate-buffered saline (pH 7.4), and rinsed twice in 50 mM Tris HCl + 0.1% BSA buffer. Slides were then put in a humid chamber and covered with 400 µL of incubation medium (50 mM Tris HCl, 0.025% bacitracin, 5 mM MgCl2, 0,1% BSA) containing the radiolabeled I [125] OVTA (Perkin Elmer), at a concentration of 10 pM. After a 2h incubation under gentle agitation, the incubation medium is removed and slides are rinsed twice in ice-cold incubation medium and a third time in ice cold distilled water. Each slide is then dried in a stream of cool air, and placed in an X-ray cassette in contact with a KODAK film for 3 days.

ROIs were chosen and analyzed through ImageJ, using Paxinos’ Mouse Brain Atlas as a reference to find the brain areas of interest. To remove background noise caused by nonspecific binding, each slide was compared with its contiguous one, which had been incubated in presence of an excess of "cold" oxytocin (2 μM). Net grey intensity was quantified and then converted to nCi/mg tissue equivalent using a calibration curve. For each region, a minimum of 4 slices per brain were included in the analysis. Data plotted on graphs are the differences between the total and the nonspecific binding. Right and left hemispheres were kept separate.

**Chromogenic In situ Hybridization**

The two probes used are synthetic oligonucleotide probes complementary to the nucleotide sequence 1198 – 2221 of Oxtr (NM_001081147.1) (Oxtr-E4-C2, ACD Cat #411101-C2) and 3229 – 4220 of Magel2 (NM_013779.2) (Magel2-01, ACD Cat #535901). Briefly, slides were fixed in 4% paraformaldehyde in PBS (pH 9.5) on ice for 2 hours and dehydrated in increasing concentrations of alcohol, then stored in 100% ethanol overnight at -20°C. The slides were air dried for 10 minutes, then pretreated in target retrieval solution (ref. 322001, ACD) for 5 minutes while boiling, after which, slides were rinsed 2 times in water followed by 100% ethanol and then air dried. A hydrophobic barrier pen (ImmEdge) was used to create a barrier around selected sections. Selected sections were then incubated with protease plus (ref. 322331, ACD) for 15 minutes in a HybEZ oven (ACD) at 40°C, followed by water washes. The sections were then hybridized with the probe mixture at 40°C for 2 hr per slide. Unbound hybridization probes were removed by washing 2 times in wash buffer. After hybridization, sections were subjected to signal amplification using the HD 2.5 detection Kit following the kit protocol. Hybridization signal was detected using a mixture of fast-RED solutions A and B (60:1) for Oxtr-E4-C2 and a mixture of Fast-GREEN solutions A and B (50:1) for Magel2-01. The slides were then counterstained with Gill’s hematoxylin and air-dried in a 60°C oven for 15 min. Slides were cooled and cover-slipped with Vectamount TM (Vector Laboratories, Inc. Burlingame, CA). Slides were imaged at 4x and 20x on a bright field microscope (Keyence BZ-X710, Keyence Corp., Osaka, Japan). Hippocampal sections were investigated for colocalization of *Oxtr* (red) with *Magel2* (blue-green) transcripts.

**Western Blot**

P7 mice were sacrificed and hippocampi were dissected and rapidly frozen in liquid nitrogen and stored at -80°C until protein extraction. Hippocampi were lysed in lysis buffer (50 mM Tris/HCl, pH 7.5, 1 mM EGTA, 1 mM EDTA, 50 mM sodium fluoride, 5 mM sodium pyrophosphate, 1 mM sodium orthovanadate, 1% (w/v) Triton-100, 0.27 M sucrose, 0.1% (v/v) 2-mercaptoethanol, and protease inhibitors (complete protease inhibitor cocktail tablets, Roche, 1 tablet per 50 mL)) and protein concentrations were determined following centrifugation of the lysate at 16,000 x g at 4 °C for 20 minutes using the Bradford method with bovine serum albumin as the standard. Tissue lysates (15 µg) in SDS sample buffer (1X NuPAGE LDS sample buffer (Invitrogen), containing 1% (v/v) 2-mercaptoethanol) were subjected to electrophoresis on polyacrylamide gels and transferred to nitrocellulose membranes. The membranes were incubated for 30 min with TBS-Tween buffer (TTBS, Tris/HCl, pH 7.5, 0.15 M NaCl and 0.2% (v/v) Tween-20) containing 5% (w/v) skim milk. The membranes were then immunoblotted in 5% (w/v) skim milk in TTBS with the indicated primary antibodies overnight at 4°C. The blots were then washed six times with TTBS and incubated for 1 hour at room temperature with secondary HRP-conjugated antibodies diluted 5000-fold in 5% (w/v) skim milk in TTBS. After repeating the washing steps, the signal was detected with the enhanced chemiluminescence reagent. Immunoblots were developed using ChemiDoc™ Imaging Systems (Bio-Rad). Primary antibodies used were: anti-KCC2 phospho-Ser940 (Thermo Fisher Scientific, cat #PA5-95678), anti-KCC2 phospho-Thr1007 (Thermo Fisher Scientific, cat #PA5-95677), anti-Pan-KCC2, residues 932-1043 of human KCC2 (NeuroMab, cat #73-013), anti(neuronal)-β-Tubulin III (Sigma-Aldrich, cat #T8578). Horseradish peroxidase-coupled (HRP) secondary antibodies used for immunoblotting were from Pierce. Figures were generated using Photoshop and Illustrator (Adobe). The relative intensities of immunoblot bands were determined by densitometry with ImageJ software.

**Statistical Analysis**

Statistical analyses were performed using GraphPad Prism (GraphPad Software, Prism 8.0 software, Inc, La Jolla, CA, USA). All statistical tests were two-tailed and the level of significance was set at P<0.05. Appropriate tests were conducted depending on the experiment; tests are indicated in the figure legends or detailed in supplementary statistical file. Values are indicated as Q2 (Q1, Q3), where Q2 is the median, Q1 is the first quartile and Q3 is the third quartile when non-parametric tests were performed and scatter dot plots report Q2 (Q1, Q3) or as mean ± SEM when parametric tests were performed usually in histograms. N refers to the number of animals or primary culture preparations, while n refers to the number of brain sections or hippocampi or cells recorded.

T-test (parametric test) or Mann-Whitney (MW) non-parametric test were performed to compare two matched or unmatched groups. One-way ANOVA or Mixed model with repeated measurements followed by Bonferroni’s or Tukey’s multiple comparison post-hoc tests were used to compare three or more independent groups. Two-way ANOVA followed by Tukey’s post-hoc test was performed to compare the effect of two factors on unmatched groups. *: p< 0.05; **: p <0.01; ***: p<0.001; ****: p<0.0001. All the statistical analyses (corresponding to each figure) are reported in a specific file.

**REFERENCES**

1 Matarazzo, V. & Muscatelli, F. Natural breaking of the maternal silence at the mouse and human imprinted Prader-Willi locus: A whisper with functional consequences. *Rare diseases* **1**, e27228, doi:10.4161/rdis.27228 (2013).

2 Schaller, F. *et al.* A single postnatal injection of oxytocin rescues the lethal feeding behaviour in mouse newborns deficient for the imprinted Magel2 gene. *Hum Mol Genet* **19**, 4895-4905, doi:10.1093/hmg/ddq424 (2010).

3 Meziane, H., Ouagazzal, A. M., Aubert, L., Wietrzych, M. & Krezel, W. Estrous cycle effects on behavior of C57BL/6J and BALB/cByJ female mice: implications for phenotyping strategies. *Genes Brain Behav* **6**, 192-200, doi:10.1111/j.1601-183X.2006.00249.x (2007).

4 Zhang, J. B. *et al.* Oxytocin is implicated in social memory deficits induced by early sensory deprivation in mice. *Molecular brain* **9**, 98, doi:10.1186/s13041-016-0278-3 (2016).

5 Kaech, S. & Banker, G. Culturing hippocampal neurons. *Nature protocols* **1**, 2406-2415, doi:10.1038/nprot.2006.356 (2006).

6 Tyzio, R. *et al.* Membrane potential of CA3 hippocampal pyramidal cells during postnatal development. *J Neurophysiol* **90**, 2964-2972, doi:10.1152/jn.00172.2003 (2003).

7 Scorcioni, R., Polavaram, S. & Ascoli, G. A. L-Measure: a web-accessible tool for the analysis, comparison and search of digital reconstructions of neuronal morphologies. *Nature protocols* **3**, 866-876, doi:10.1038/nprot.2008.51 (2008).
