# Supplementary material for: Oxytocin administration in neonates shapes hippocampal circuitry and restores social behavior in a mouse model of autism
Source: Mol Psychiatry. 2021 Jul 21;26(12):7582–95. doi: 10.1038/s41380-021-01227-6 (PMC8872977; doi:10.1038/s41380-021-01227-6)
Supplement: Supplementary file 2 — Supplemental Information - Statistical Tables [file 41380_2021_1227_MOESM2_ESM.pdf]

Supplementary Table 1:
Social behavior in the 3-chamber test of adult male WT vs Magel2 KO and adult male WT or Magel2 KO vehicle-treated or OT-treated as neonates

**Figure**	**Parameter**	**Test specifications**		**Genotype**	**Treatment**	**Sex**	**Number of individuals**	**Mean ± SEM**	**Statistical test**	**p-value**
Fig2 B	Interaction time (sniffing in seconds)	Social exploration	S1	WT	-	male	12	78.0 ± 7.7	paired t-test	**p=0.00134**
			Empty					34.5 ± 6.6		
		Social discrimination	S1	WT	-	male		33.7 ± 4.7		**p=0.000129**
			S2					70.7 ± 7.4		
		Short-term social memory	S1	WT	-	male		28.0 ± 6.0		**p=0.0263**
			S3					77.2 ± 16.3		
		Social exploration	S1	*Magel2* KO	-	male	9	68.1 ± 6.8		**p=0.000133**
			Empty					31.5 ± 5.0		
		Social discrimination	S1	*Magel2* KO	-	male		30.0 ± 4.3		**p=0.0169**
			S2					61.0 ± 7.6		
		Short-term social memory	S1	*Magel2* KO	-	male		38.2 ± 6.4		p=0.472
			S3					43.9 ± 7.1		
Fig2 C	Interaction time (sniffing in seconds)	Social exploration	S1	WT	vehicle	male	18	83.5 ± 6.5	paired t-test	**p=0.00179**
			Empty					49.2 ± 5.6		
		Social discrimination	S1	WT	vehicle	male		44.2 ± 3.8		**p=0.000291**
			S2					84.5 ± 6.9		
		Short-term social memory	S1	WT	vehicle	male		34.0 ± 3.3		**p=0.000571**
			S3					67.1 ± 6.4		
		Social exploration	S1	WT	OT	male	10	88.3 ± 6.3		**p<0.0001**
			Empty					42.0 ± 2.3		
		Social discrimination	S1	WT	OT	male		49.9 ± 4.9		**p=0.00776**
			S2					84.2 ± 10.5		
		Short-term social memory	S1	WT	OT	male		36.4 ± 3.4		**p=0.00917**
			S3					68.8 ± 7.4		
Fig2 D	Interaction time (sniffing in seconds)	Social exploration	S1	*Magel2* KO	vehicle	male	19	65.8 ± 5.1	paired t-test	**p<0.0001**
			Empty					31.0 ± 3.2		
		Social discrimination	S1	*Magel2* KO	vehicle	male		29.5 ± 4.3		**p<0.0001**
			S2					73.5 ± 7.0		
		Short-term social memory	S1	*Magel2* KO	vehicle	male		38.8 ± 4.0		p=0.259
			S3					44.2 ± 3.1		
		Social exploration	S1	*Magel2* KO	OT	male	19	89.1 ± 6.0		**p<0.0001**
			Empty					36.2 ± 4.5		
		Social discrimination	S1	*Magel2* KO	OT	male		34.0 ± 4.7		**p<0.0001**
			S2					87.2 ± 6.2		
		Short-term social memory	S1	*Magel2* KO	OT	male		32.4 ± 3.8		**p=0.000702**
			S3					58.6 ± 6.5		

Supplementary Table 2:
cFos activity in aCA2/CA3d and aDG regions of Magel2 KO and WT adult male mice following the social memory task in the 3-chamber test

Figure	Parameter	Brain region	Genotype	Behavioral test	Treatment	Sex	Number of individuals	Number of sections	Mean ± SEM	Statistical test	Fixed effects F(DFn,DFd)	p-value
Fig3 D	Number of cFos-positive cells	aCA2/CA3d	WT	-SI	-	male	5	40	27.70 ± 1.21	Mixed model with repeated measurements, Tukey's multiple comparison test	Effect of sections F(7,101)=0.5567; p=0.78<br><br>Effect group of mice F(1.52,51.32)=61.68; **p<0.0001**	WT-SI vs Magel2KO-SI, p=0.2725<br>**WT-SI vs WT+SI, p<0.0001**<br>**WT-SI vs Magel2KO+SI, p<0.0001**<br>**Magel2KO-SI vs WT+SI, p<0.0001**<br>**Magel2KO-SI vs Magel2KO+SI, p<0.0001**<br>WT+SI vs Magel2KO+SI, p=0.0525
			WT	+SI	-	male	4	31	46.48 ± 2.15			
			Magel2 KO	-SI	-	male	4	32	24.63 ± 0.82			
			Magel2 KO	+SI	-	male	4	30	60.18 ± 3.07			
Fig3 E	Number of cFos-positive cells	aDG	WT	-SI	-	male	5	40	40.980 ± 1.16	Mixed model with repeated measurements, Tukey's multiple comparison test	Effect of sections F(7,100)=0.6139; p=0.74<br><br>Effect group of mice F(1.456,48.52)=44.63; **p<0.0001**	WT-SI vs Magel2KO-SI, p=0.8603<br>**WT-SI vs WT+SI, p<0.0001**<br>**WT-SI vs Magel2KO+SI, p<0.0001**<br>**Magel2KO-SI vs WT+SI, p<0.0001**<br>**Magel2KO-SI vs Magel2KO+SI, p<0.0001**<br>WT+SI vs Magel2KO+SI, p=0.9620
			WT	+SI	-	male	4	30	71.63 ± 3.69			
			Magel2 KO	-SI	-	male	4	32	39.66 ± 1.33			
			Magel2 KO	+SI	-	male	4	30	74.05 ± 2.19			

Supplementary Table 3:
Quantification of OT binding sites by brain autoradiography in the adult hippocampus of Magel2 KO male mice treated with OT or vehicle versus WT-vehicle male mice

Figure	Parameter	Brain region	Genotype	Treatment	Sex	Number of individuals	Number of sections	Mean ± SEM	Statistical test	Fixed effects F(DFn,DFd)	p-value
Fig4 B	nCi/mg of tissue equivalent	aCA2/CA3	WT	vehicle	male	3	24	0.0747 ± 0.005	one-way ANOVA, Bonferroni's post-hoc test	Effect group of mice F(2,14)=19.70; **p<0.0001**	**WT-vehicle vs *Magel2* KO-vehicle, p<0.0001**
			Magel2 KO	vehicle	male	3	24	0.1404 ± 0.009			**WT-vehicle vs *Magel2* KO+OT, p=0.0021**
			Magel2 KO	OT	male	3	24	0.1208 ± 0.007			*Magel2* KO-vehicle vs *Magel2* KO+OT, p=0.2232
Fig4 D	nCi/mg of tissue equivalent	aDG	WT	vehicle	male	3	24	0.0800 ± 0.007	one-way ANOVA, Bonferroni's post-hoc test	Effect group of mice F(2,15)=25.34; **p<0.0001**	**WT-vehicle vs *Magel2* KO-vehicle, p<0.0001**
			Magel2 KO	vehicle	male	3	24	0.1404 ± 0.005			WT-vehicle vs *Magel2* KO+OT, p>0.9999
			Magel2 KO	OT	male	3	24	0.0796 ± 0.008			***Magel2* KO-vehicle vs *Magel2* KO+OT, p<0.0001**
Fig4 F	nCi/mg of tissue equivalent	vCA1/CA2/CA3	WT	vehicle	male	3	24	0.1679 ± 0.006	one-way ANOVA, Bonferroni's post-hoc test	Effect group of mice F(2,13)=4.47; p=0.033	WT-vehicle vs *Magel2* KO-vehicle, p=0.0722
			Magel2 KO	vehicle	male	3	24	0.1299 ± 0.006			WT-vehicle vs *Magel2* KO+OT, p=0.0590
			Magel2 KO	OT	male	3	24	0.1300 ± 0.011			*Magel2* KO-vehicle vs *Magel2* KO+OT, p>0.9999

Supplementary Table 4:

Quantification of somatostatin (SST) immunopositive cells in the anterior hippocampus region of adult *Magel2* KO, WT, *Magel2* KO treated with OT and WT-vehicle male mice

**Figure**	**Parameter**	**Brain region**	**Genotype**	**Treatment**	**Sex**	**Number of individuals**	**Number of sections**	**Mean ± SEM**	**Statistical test**	**Fixed effects F(DFn,DFd)**	**p-value**
Fig5 M	Number of SST-positive cells	aCA2/CA3d	WT	-	male	4	56	48.96 ± 2.34	Mixed model with repeated measurements, Tukey's multiple comparison test	Effect of sections F(13,156)=1.098; p=0.36<br>Effect group of mice F(1.937,100.7)=90.88; **p<0.0001**	**WT vs Magel2 KO, p<0.0001**<br>WT vs WT-vehicle, p=0.7505<br>**WT vs Magel2 KO+OT, p=0.0177**<br>**Magel2 KO vs WT-vehicle, p<0.0001**<br>**Magel2 KO vs Magel2 KO+OT, p<0.0001**<br>**WT-vehicle vs Magel2 KO+OT, p=0.0120**
			Magel2 KO	-	male	4	55	93.67 ± 3.58			
			WT	vehicle	male	3	37	46.37 ± 2.30			
			Magel2 KO	OT	male	5	64	40.08 ± 1.00			

Fig5 N	Number of SST-positive cells	aDG	WT	-	male	4	56	22.39 ± 1.13	Mixed model with repeated measurements, Tukey's multiple comparison test	Effect of sections F(13,157)=0.7205; p=0.74<br>Effect group of mice F(2.175,113.8)=117.3; **p<0.0001**	**WT vs Magel2 KO, p<0.0001**<br>WT vs WT-vehicle, p=0.9534<br>WT vs Magel2 KO+OT, p=0.0708<br>**Magel2 KO vs WT-vehicle, p<0.0001**<br>**Magel2 KO vs Magel2 KO+OT, p<0.0001**<br>**WT-vehicle vs Magel2 KO+OT, p<0.0001**
			Magel2 KO	-	male	4	55	45.41 ± 1.50			
			WT	vehicle	male	3	38	23.24 ± 0.52			
			Magel2 KO	OT	male	5	64	18.81 ± 0.48			

Supplementary Table 5:

Spontaneous Glutamatergic and GABAergic synaptic activity of CA3 pyramidal neurons in the anterior hippocampus of Magel2 KO vs WT juvenile mice with or without OT-treatment

Figure	Parameter	Genotype	Treatment	Sex	Number of individuals	Number of neurons	Median (Q1,Q3)	Statistical test	Comparison	P-value
Fig6 C	Glut amplitude (pA)	WT	-	male	7	15	42(18, 61)	One way ANOVA + Tukey post-hoc test	WT vs *Magel2* KO	p<0.05
		Magel2 KO	-	male	7	16	24 (22, 34)		*Magel2* KO vs KO+OT	n.s.
		WT	OT	male	4	15	25.5 (21.7, 34)		WT vs WT+OT	p<0.01
		Magel2 KO	OT	male	5	20	27.3 (19.3, 32)		WT+OT vs KO+OT	p<0.01
Fig6 D	Glut frequency (Hz)	WT	-	male	7	15	26 (18, 35)	One way ANOVA + Tukey post-hoc test	WT vs *Magel2* KO	n.s.
		Magel2 KO	-	male	7	18	32 (27, 37.5)		*Magel2* KO vs KO+OT	p<0.01
		WT	OT	male	4	15	8.5 (2.7, 16.2)		WT vs WT+OT	p<0.01
		Magel2 KO	OT	male	5	21	11 (17.6, 23.2)		WT+OT vs KO+OT	p<0.01
Fig6 E	GABA frequency (Hz)	WT	-	male	7	15	13 (5.2, 21)	One way ANOVA + Tukey post-hoc test	WT vs *Magel2* KO	p<0.01
		Magel2 KO	-	male	7	18	23.5 (21.7, 29.2)		*Magel2* KO vs KO+OT	p<0.01
		WT	OT	male	4	14	11.5 (8, 16.2)		WT vs WT+OT	p<0.01
		Magel2 KO	OT	male	5	21	12.5 (10.5, 17.2)		WT+OT vs KO+OT	n.s.
Fig6 F	GABA amplitude (pA)	WT	-	male	7	15	29 (23.6, 33)	One way ANOVA + Tukey post-hoc test	WT vs *Magel2* KO	n.s.
		Magel2 KO	-	male	7	16	24.5 (17.5, 37.5)		*Magel2* KO vs KO+OT	n.s.
		WT	OT	male	4	15	25 (17.4, 30.4)		WT vs WT+OT	n.s.
		Magel2 KO	OT	male	5	21	27.1 (24, 31.5)		WT+OT vs KO+OT	n.s.

Supplementary Table 6:

The excitatory-to-inhibitory developmental GABA-shift in Magel2 KO versus WT hippocampi and the effect on an OT-treatment. Abundance and phosphorylation state of KCC2 in WT and Magel2 KO pups

**Figure**	**Parameter**	**Days in vitro**	**Genotype**	**Treatment**	**Number of preparations**	**Number of neurons**	**Mean ± SEM**	**Statistical test**	**p-value**
Fig7 A	Percentage of responsive neurons	DIV2	WT	-	3	169	57.88 ± 6.866	Unpaired t test with Welch's correction	p=0.479
			Magel2 KO	-	4	210	64.15 ± 5.519		
		DIV4	WT	-	5	330	29.42 ± 4.315		**p<0.0001**
			Magel2 KO	-	3	175	62.09 ± 5.196		
		DIV8	WT	-	3	206	2.553 ± 1.052		p=0.233
			Magel2 KO	-	2	179	1.016 ± 0.709		
		DIV11	WT	-	2	92	1.759 ± 1.188		p=0.813
			Magel2 KO	-	2	97	1.333 ± 1.333		

**Figure**	**Parameter**	**Age**	**Genotype**	**Treatment**	**Number of individuals**	**Number of neurons**	**Mean ± SEM**	**Statistical test**	**p-value**
Fig7 C	Driving Force GABA (mV)	P1	WT	-	3	19	12.7 ± 1.5	Unpaired t test with Welch's correction	p=0.0535
			Magel2 KO	-	3	20	16.8 ± 1.4		
		P7	WT	-	6	42	5.0 ± 1.4		**p=0.0358**
			Magel2 KO	-	7	56	8.9 ± 1.2		
		P15	WT	-	3	23	2.7 ± 1.6		p=0.6805
			Magel2 KO	-	4	29	1.9 ± 1.1		

Fig7 D	Driving Force GABA (mV)	P7	WT	vehicle	3	37	8.5 ± 1.3	one-way ANOVA, Dunnett's post-hoc test	
			WT	OT	3	37	3.5 ± 1.2		**WT-vehicle vs WT+OT, p=0.0041**
			Magel2 KO	OT	4	56	4.1 ± 0.8		**WT-vehicle vs *Magel2* KO+OT, p=0.0055**

**Figure**	**Parameter**	**Ratio**	**Genotype**	**Treatment**	**Sex**	**Number of individuals (brains)**	**Median (Q1, Q3)**	**Statistical test**	**p-value**
Fig7 F	Ratio of protein quantity	KCC2/tubulin	WT	-	male	5	5 (4.8, 5.1)	Mann Whitney	n.s.
			Magel2 KO	-	male	6	4.9 (4.5, 5)		
		P-Ser^{940}/ KCC2	WT	-	male	5	0.42 (0.21, 0.54)		**p<0,05**
			Magel2 KO	-	male	6	0.16 (0.14, 0.20)		
		P-Thr^{1007}/ KCC2	WT	-	male	5	0.36 (0.34, 0.66)		n.s.
			Magel2 KO	-	male	6	0.49 (0.40, 0.50)		

Supplementary Table S1:
Social-index values of adult male Magel2 KO vs WT and adult male Magel2 KO+OT vs Magel2 KO-vehicle mice

**Figure**	**Parameter**	**Test specifications**		**Genotype**	**Treatment**	**Sex**	**Number of individuals**	**Median (Q1,Q3)**	**Statistical test**	**p-value**
Supp Fig1 A	Social index	Social exploration index	S1/Empty	WT	-	male	9	69.129 (62.504,84.591)	Mann-Whitney	p=0.930
				Magel2 KO	-		9	70.387 (63.281,74.867)		
		Discrimination index	S2/S1	WT	-	male	9	69.399 (58.508,76.801)		p=0.596
				Magel2 KO	-		9	67.067 (51.376,76.300)		
		Short-term memory index	S3/S1	WT	-	male	9	80.484 (78.511,84.959)		**p=0.001**
				Magel2 KO	-		9	54.192 (44.777,64.752)		

**Figure**	**Parameter**	**Test specifications**		**Genotype**	**Treatment**	**Sex**	**Number of individuals**	**Median (Q1,Q3)**	**Statistical test**	**p-value**
Supp Fig1 B	Social index	Social exploration index	S1/Empty	*Magel2* KO	vehicle	male	19	71.333 (65.560,75.758)	Mann-Whitney	p=0.484
				Magel2 KO	OT		19	75.079 (58.744,82.863)		
		Discrimination index	S2/S1	*Magel2* KO	vehicle	male	19	72.195 (61.802,83.006)		p=0.907
				Magel2 KO	OT		19	72.581 (60.550,81.239)		
		Short-term memory index	S3/S1	*Magel2* KO	vehicle	male	19	51.515 (45.474,60.818)		**p=0.021**
				Magel2 KO	OT		19	65.056 (55.882,72.986)		

Supplementary Table S2:
Behavioral tests in adult male and female WT mice vehicle-treated or OT-treated as neonates

Figure	Parameter	Test specifications		Genotype	Treatment	Sex	Number of individuals	Median (Q1,Q3)	Statistical test	p-value
Supp Fig2 A	NOR	Discrimination index (% of preference)	same object	WT	vehicle	male	10	46.822 (42.071,53.412)	One-Sample Singled Rank Test 50%	p=0.557
			new object				10	76.740 (64.570,83.103)		**p=0.002**
			same object	WT	OT	male	10	52.327 (39.516,59.132)		p=1.000
			new object				10	65.772 (59.652,73.432)		**p=0.002**
		Discrimination index (% of preference)	same object	WT	vehicle	female	19	46.334 (41.667,55.738)	One-Sample Singled Rank Test 50%	p=0.293
			new object				17	65.625 (57.805,69.979)		**p<0.001**
			same object	WT	OT	female	12	46.392 (38.371,48.776)		**p=0.034**
			new object				12	64.978 (56.382,74.646)		**p<0.001**
Supp Fig2 B	Open Field	Distance mouved (in minutes)		WT	vehicle	male	18	26.517 (23.086,32.118)	Mann-Whitney	p=0.111
				WT	OT		11	21.989 (19.289,26.250)		
				WT	vehicle	female	16	31.654 (23.711,37.174)		**p=0.048**
				WT	OT		12	22.620 (15.569,32.495)		
		Rearing (number of events)		WT	vehicle	male	18	51.000 (36.000,62.000)		p=0.747
				WT	OT		11	52.000 (41.250,60.750)		
				WT	vehicle	female	16	41.000 (27.000,45.000)		p=0.185
				WT	OT		12	47.000 (38.000,60.000)		
		Grooming time (in seconds)		WT	vehicle	male	18	36.160 (25.120,47.280)	Mann-Whitney	p=0.723
				WT	OT		11	45.680 (17.140,65.420)		
				WT	vehicle	female	16	25.600 (21.600,45.120)		p=0.299
				WT	OT		12	36.400 (21.080,53.600)		
		Time in zone		WT	vehicle	male	18	28.616 (26.283,38.172)		p=0.574
				WT	OT		11	31.249 (28.050,36.235)		
				WT	vehicle	female	16	27.970 (21.667,31.806)		p=0.676
				WT	OT		12	25.530 (12.785,42.204)		
Supp Fig2 C	EPM	Time in open-arms		WT	vehicle	male	21	18.960 (14.680,30.320)	Mann-Whitney	p=0.342
				WT	OT		10	23.600 (17.440,40.840)		
				WT	vehicle	female	19	25.440 (16.720,44.800)		p=0.491
				WT	OT		12	31.720 (21.400,42.540)		
		Open-arms entries		WT	vehicle	male	21	10.000 (6.500,11.500)	Mann-Whitney	p=0.149
				WT	OT		10	12.000 (7.000,14.250)		
				WT	vehicle	female	19	12.000 (11.000,15.000)		p=0.326
				WT	OT		12	14.000 (11.250,19.500)		

Supplementary Table S3:
Social behavior in 3-chamber test of adult female WT not treated or vehicle-treated or OT-treated as neonates

Figure	Parameter	Test specifications		Genotype	Treatment	Sex	Number of individuals	Mean ± SEM	Statistical test	p-value
Supp Fig3 B	Interaction time (sniffing in seconds)	Social exploration	S1	WT	-	female	11	85.18 ± 11.89	paired t-test	**p=0.0201**
			Empty					48.85 ± 8.04		
		Social discrimination	S1	WT	-	female		48.37 ± 5.71		**p=0.0329**
			S2					73.49 ± 10.53		
		Short-term social memory	S1	WT	-	female		43.99 ± 4.82		p=0.488
			S3					50.54 ± 8.99		

Figure	Parameter	Test specifications		Genotype	Treatment	Sex	Number of individuals	Mean ± SEM	Statistical test	p-value
Supp Fig3 C	Interaction time (sniffing in seconds)	Social exploration	S1	WT	vehicle	female	14	75.95 ± 6.60	paired t-test	**p=0.0016**
			Empty					42.26 ± 6.61		
		Social discrimination	S1	WT	vehicle	female		48.69 ± 5.90		p=0.0607
			S2					74.41 ± 8.51		
		Short-term social memory	S1	WT	vehicle	female		37.68 ± 4.75		p=0.4414
			S3					44.86 ± 7.20		
		Social exploration	S1	WT	OT	female	10	103.3 ± 12.06		**p=0.0033**
			Empty					43.70 ± 5.24		
		Social discrimination	S1	WT	OT	female		40.09 ± 6.10		**p=0.0163**
			S2					75.80 ± 8.38		
		Short-term social memory	S1	WT	OT	female		39.10 ± 5.02		p=0.1615
			S3					66.38 ± 17.68		

Supplementary Table S4:
Quantification of parvalbumin (PV) immunopositive cellsin the anterior hippocampus of adult *Magel2* KO and WT male mice

**Figure**	**Parameter**	**Brain region**	**Genotype**	**Treatment**	**Sex**	**Number of individuals**	**Number of sections**	**Mean ± SEM**	**Statistical test**	**Fixed effects F(DFn,DFd)**
Supp Fig4 E	Number of PV-positive cells	aCA2/CA3d	WT	-	male	4	18	7.50 ± 0.47	Mixed model with repeated measurements	Effect of sections F(2.082,10.41)=0.467; p=0.6469
			Magel2 KO	-	male	4	18	7.28 ± 0.78		Effect group of mice F(1,6)=0.0007; p=0.9799

Supp Fig4 F	Number of PV-positive cells	aDG	WT	-	male	4	18	5.95 ± 0.84	Mixed model with repeated measurements	Effect of sections F(1.284,6.418)=0.353; p=0.6254
			Magel2 KO	-	male	4	18	4.05 ± 0.52		Effect group of mice F(1,6)=0.9862; p=0.3590

Supplementary Table S5:

Miniatures Glutamatergic and GABAergic synaptic activity of aCA3 pyramidal neurons in the anterior hippocampus of *Magel2* KO vs WT juvenile mice

**Figure**	**Parameter**	**Genotype**	**Treatment**	**Sex**	**Number of individuals**	**Number of neurons**	**Mean ± SEM**	**Statistical test**	**p-value**
Supp Fig5 B	Glut amplitude (pA)	WT	-	male	5	13	33.3 ± 3	Mann-Whitney	**p<0,01**
		Magel2 KO	-	male	5	14	23.8 ± 1.8		
Supp Fig5 C	Glut frequency (Hz)	WT	-	male	5	13	0.73 ± 0.2	Mann-Whitney	n.s.
		Magel2 KO	-	male	5	16	0.67 ± 0.14		
Supp Fig5 D	GABA amplitude (pA)	WT	-	male	5	13	17.6 ± 2.7	Mann-Whitney	n.s.
		Magel2 KO	-	male	5	11	13.5 ± 1		
Supp Fig5 E	GABA frequency (Hz)	WT	-	male	5	12	0.30 ± 0.08	Mann-Whitney	n.s.
		Magel2 KO	-	male	5	12	0.26 ± 0.03		

Supplementary Table S6:

Morphology of aCA3 recorded pyramidal neurons in Magel2 KO vs WT mice

Figure	Parameter	Genotype	Treatment	Sex	Number of individuals	Number of neurons	Median (Q1,Q3)	Statistical test	p-value
Supp Fig6 C	Total length apical dendrites	WT	-	male	3	7	3957 (3349,4372)	Mann-Whitney	p=0.89
		Magel2 KO	-	male	3	11	3982 (3472,4760)		
Supp Fig6 D	Total length basal dendrites	WT	-	male	3	7	4914 (4102,5739)	Mann-Whitney	p=0.46
		Magel2 KO	-	male	3	11	5596 (4546,6318)		
Supp Fig6 E	Mean number of apical bifurcations	WT	-	male	3	7	30 (22,36)	Mann-Whitney	p=0.31
		Magel2 KO	-	male	3	11	32 (31,37)		
Supp Fig6 F	Mean number of basal bifurcations	WT	-	male	3	7	35 (30,38)	Mann-Whitney	p=0.25
		Magel2 KO	-	male	3	11	30 (25,33)		
Supp Fig6 G	Mean number of apical branches	WT	-	male	3	7	61 (45,74)	Mann-Whitney	p=0.31
		Magel2 KO	-	male	3	11	65 (64,75)		
Supp Fig6 H	Mean number of basal branches	WT	-	male	3	7	74 (64,80)	Mann-Whitney	p=0.29
		Magel2 KO	-	male	3	11	64 (70,84)		

Supplementary Table S7:
Parameters to validate the in vitro Calcium imaging analysis and the DFGABA study in Magel2 KO vs WT mice

Figure	Parameter	Days in vitro	Genotype	Treatment	Number of preparations	Number of neurons	Mean ± SEM	Statistical test	p-value
Supp Fig7 A	Amplitude of GABA-induced peaks	DIV2	WT	-	3	169	0.108 ± 0.007	Unpaired t test with Welch's correction	p=0.873
			Magel2 KO	-	4	210	0.106 ± 0.006		
		DIV4	WT	-	5	330	0.101 ± 0.006		p=0.251
			Magel2 KO	-	3	175	0.112 ± 0.007		
		DIV8	WT	-	3	206	0.076 ± 0.024		p=0.53
			Magel2 KO	-	2	179	0.121 ± 0.051		
		DIV11	WT	-	2	92	0.047 ± 0.004		
			Magel2 KO	-	2	97	0.041		
Supp Fig7 B	Amplitude of KCl-induced peaks	DIV2	WT	-	3	169	0.261 ± 0.011	Unpaired t test with Welch's correction	**p=0.007**
			Magel2 KO	-	4	210	0.223 ± 0.009		
		DIV4	WT	-	5	330	0.309 ± 0.010		**p=0.0059**
			Magel2 KO	-	3	175	0.271 ± 0.010		
		DIV8	WT	-	3	206	0.691 ± 0.024		**p<0.0001**
			Magel2 KO	-	2	179	0.468 ± 0.017		
		DIV11	WT	-	2	92	0.382 ± 0.021		**p=0.009**
			Magel2 KO	-	2	97	0.463 ± 0.022		

Figure	Parameter	Age	Genotype	Treatment	Number of individuals	Number of neurons	Mean ± SEM	Statistical test	p-value
Supp Fig7 C	Driving Force GABA	P7 - interneurons	WT	-	4	12	7.9 ± 1.4	Unpaired t test with Welch's correction	p=0.8150
			Magel2 KO	-	4	17	8.5 ± 2.3		
		P7 - pyramidal cells	WT	-	6	42	5.0 ± 1.4		**p=0.0358**
			Magel2 KO	-	7	54	8.9 ± 1.2		
Supp Fig7 D	Resting Membrane Potential	P7 - interneurons	WT	-	4	15	-69.6 ± 2.2	Unpaired t test with Welch's correction	p=0.1198
			Magel2 KO	-	4	20	-65.2 ± 1.6		
		P7 - pyramidal cells	WT	-	4	22	-69.0 ± 1.4		p=0.4346
			Magel2 KO	-	4	24	-67.1 ± 1.9		
Supp Fig7 E	Capacitance	P7 - interneurons	WT	-	4	7	53.7 ± 7.0	Unpaired t test with Welch's correction	p=0.5908
			Magel2 KO	-	4	14	59.4 ± 7.5		
		P7 - pyramidal cells	WT	-	4	22	87.7 ± 14.3		p=0.2179
			Magel2 KO	-	4	25	68.1 ± 6.0		
Supp Fig7 F	Conductance	P7 - interneurons	WT	-	4	12	12.3 ± 1.2	Unpaired t test with Welch's correction	p=0.8115
			Magel2 KO	-	4	16	12.6 ± 0.9		
		P7 - pyramidal cells	WT	-	4	24	12.4 ± 0.9		p=0.7526
			Magel2 KO	-	4	30	12.1 ± 0.6		
