# Supplementary figures and images for: Oxytocin administration in neonates shapes hippocampal circuitry and restores social behavior in a mouse model of autism
Source: Mol Psychiatry. 2021 Jul 21;26(12):7582–95. doi: 10.1038/s41380-021-01227-6 (PMC8872977; doi:10.1038/s41380-021-01227-6)

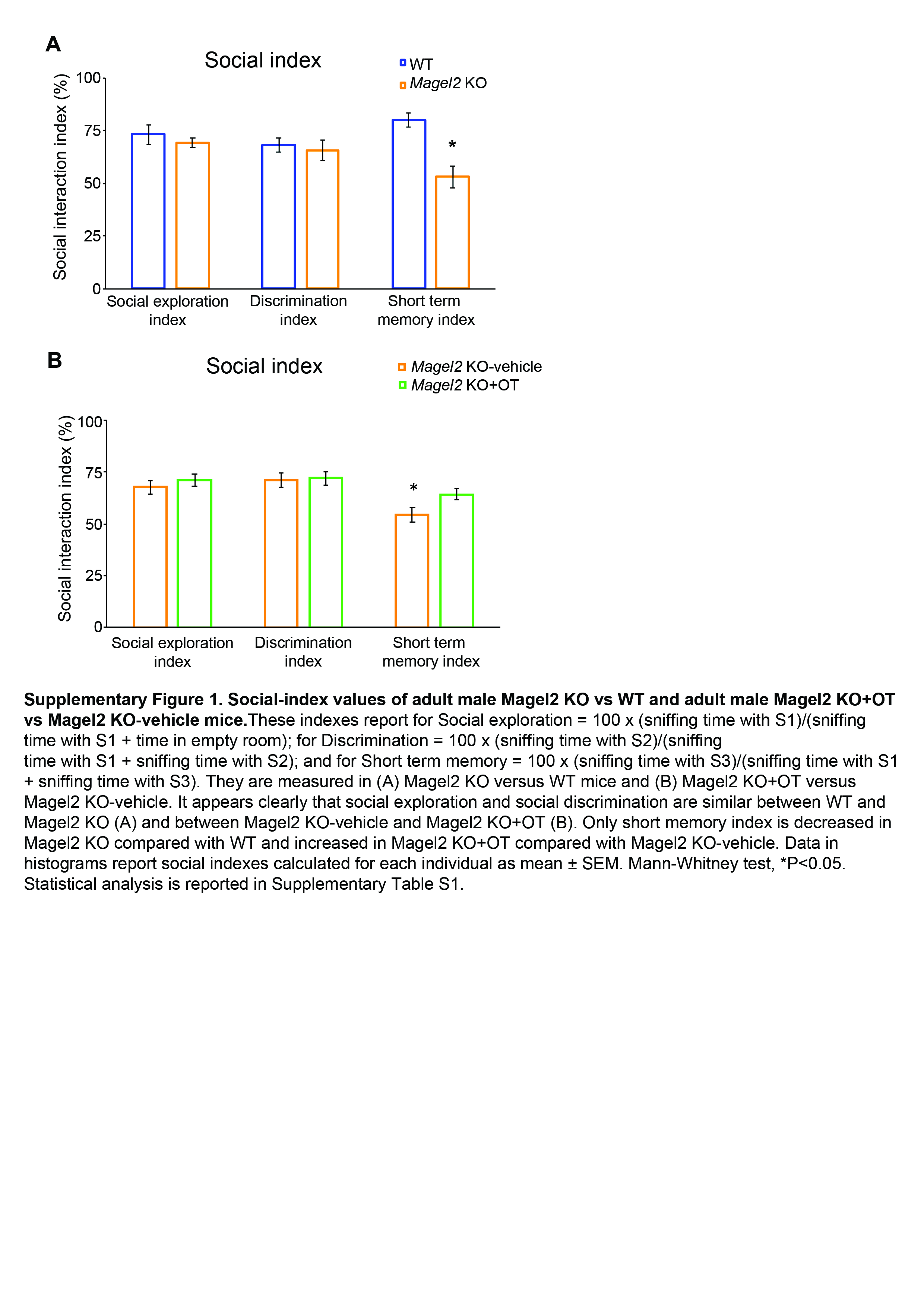

Supplement: Supplementary file 3 — Supplementary Figure 1 [file 41380_2021_1227_MOESM3_ESM.tif]

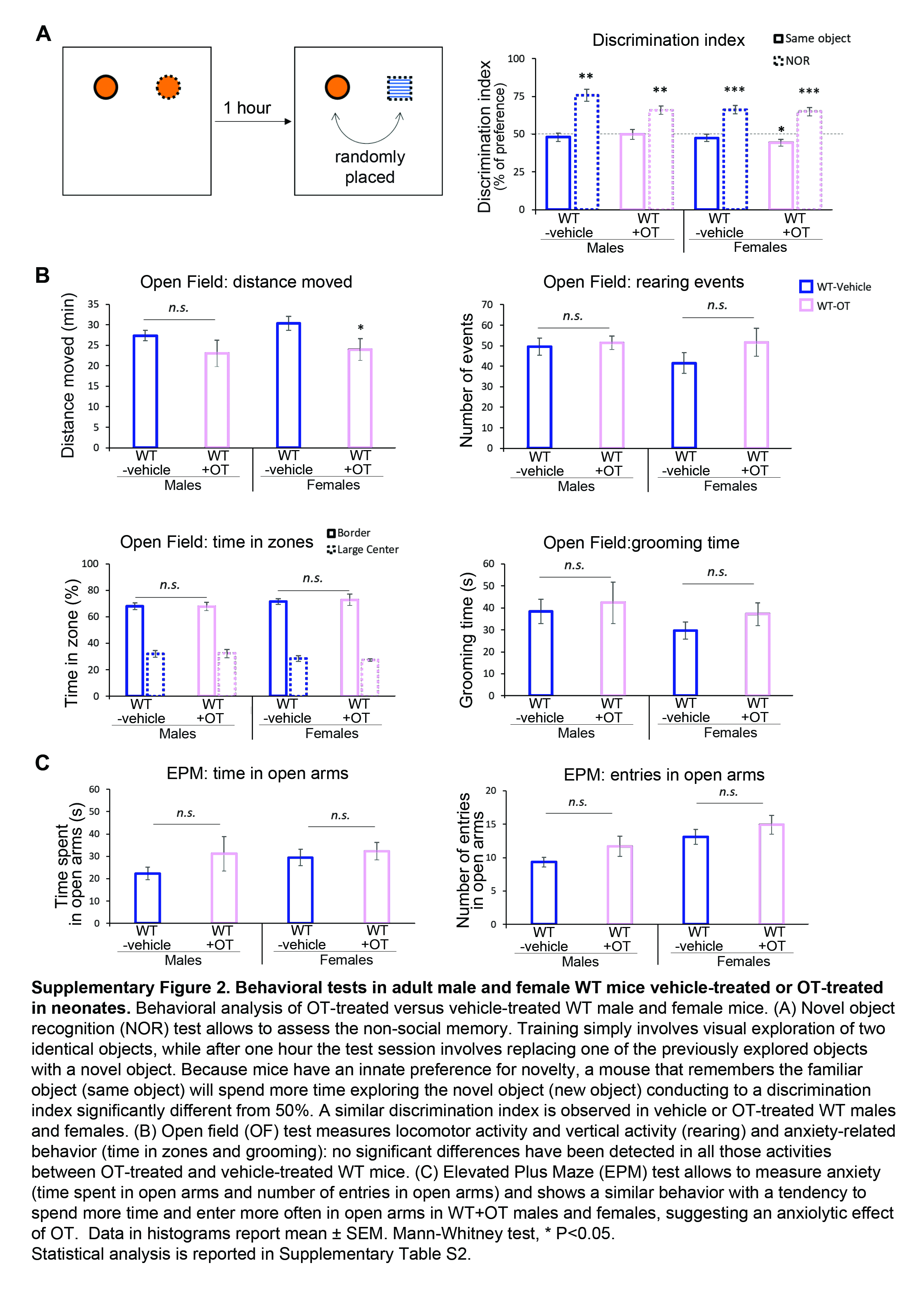

Supplement: Supplementary file 4 — Supplementary Figure 2 [file 41380_2021_1227_MOESM4_ESM.tif]

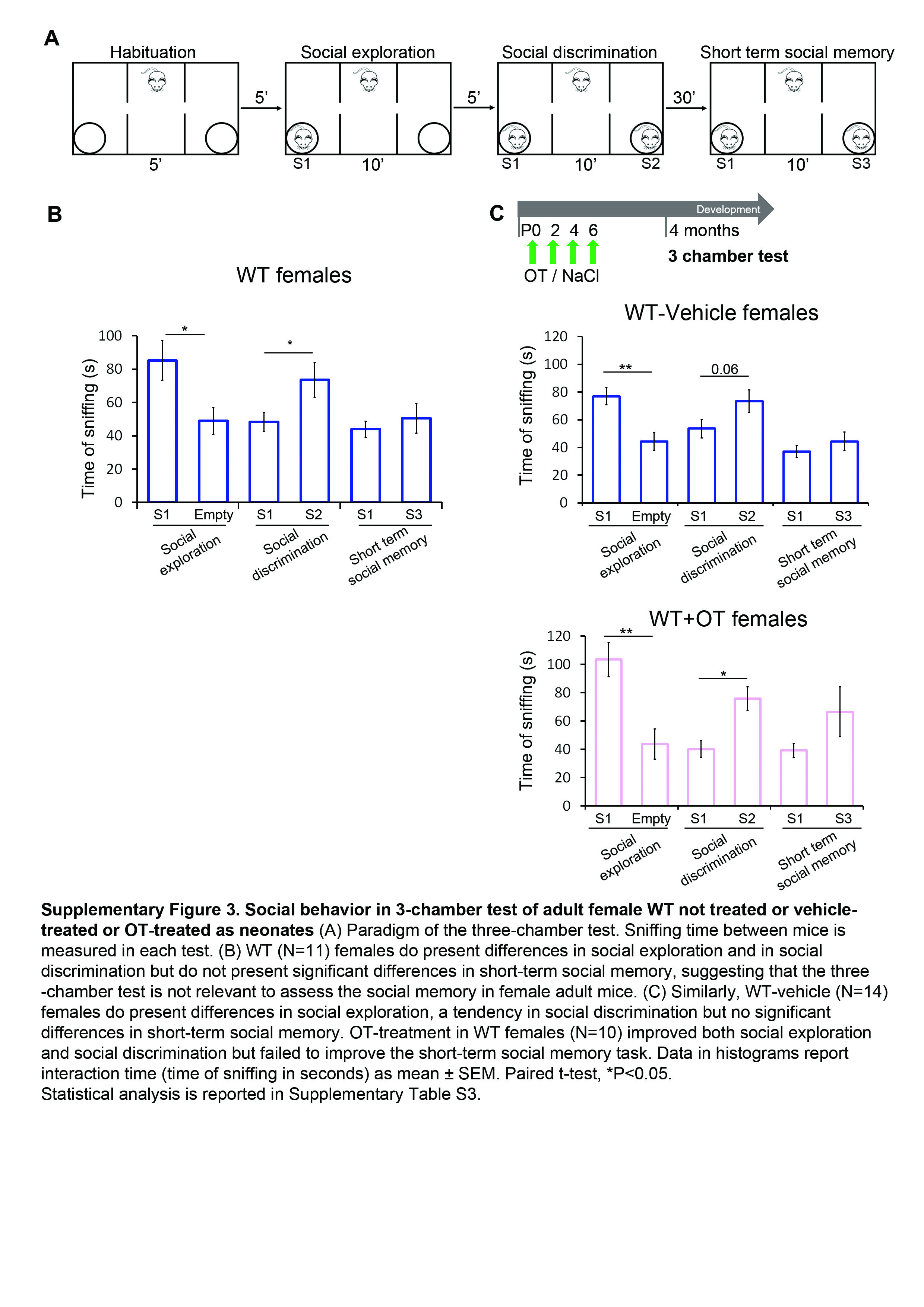

Supplement: Supplementary file 5 — Supplementary Figure 3 [file 41380_2021_1227_MOESM5_ESM.tif]

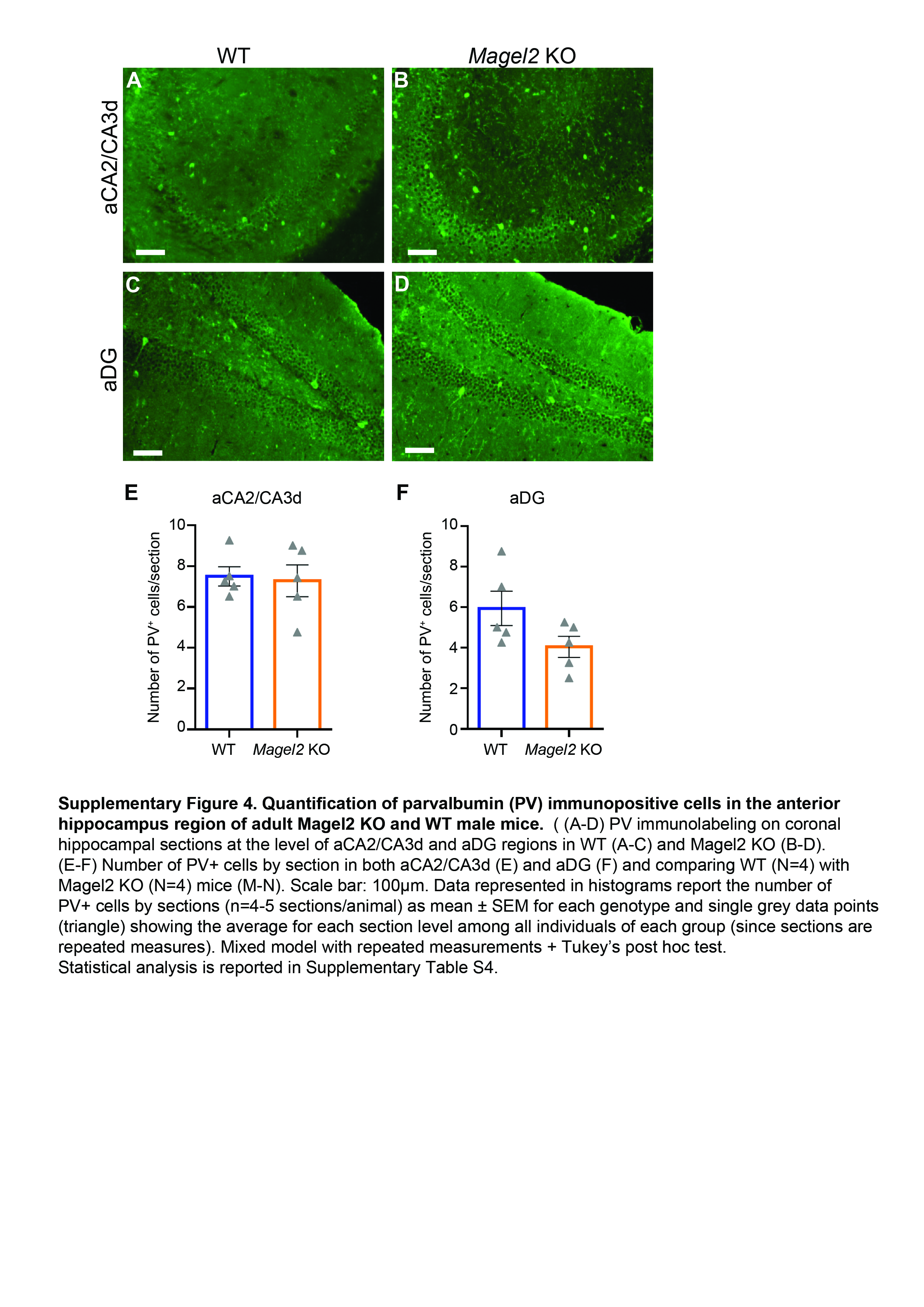

Supplement: Supplementary file 6 — Supplementary Figure 4 [file 41380_2021_1227_MOESM6_ESM.tif]

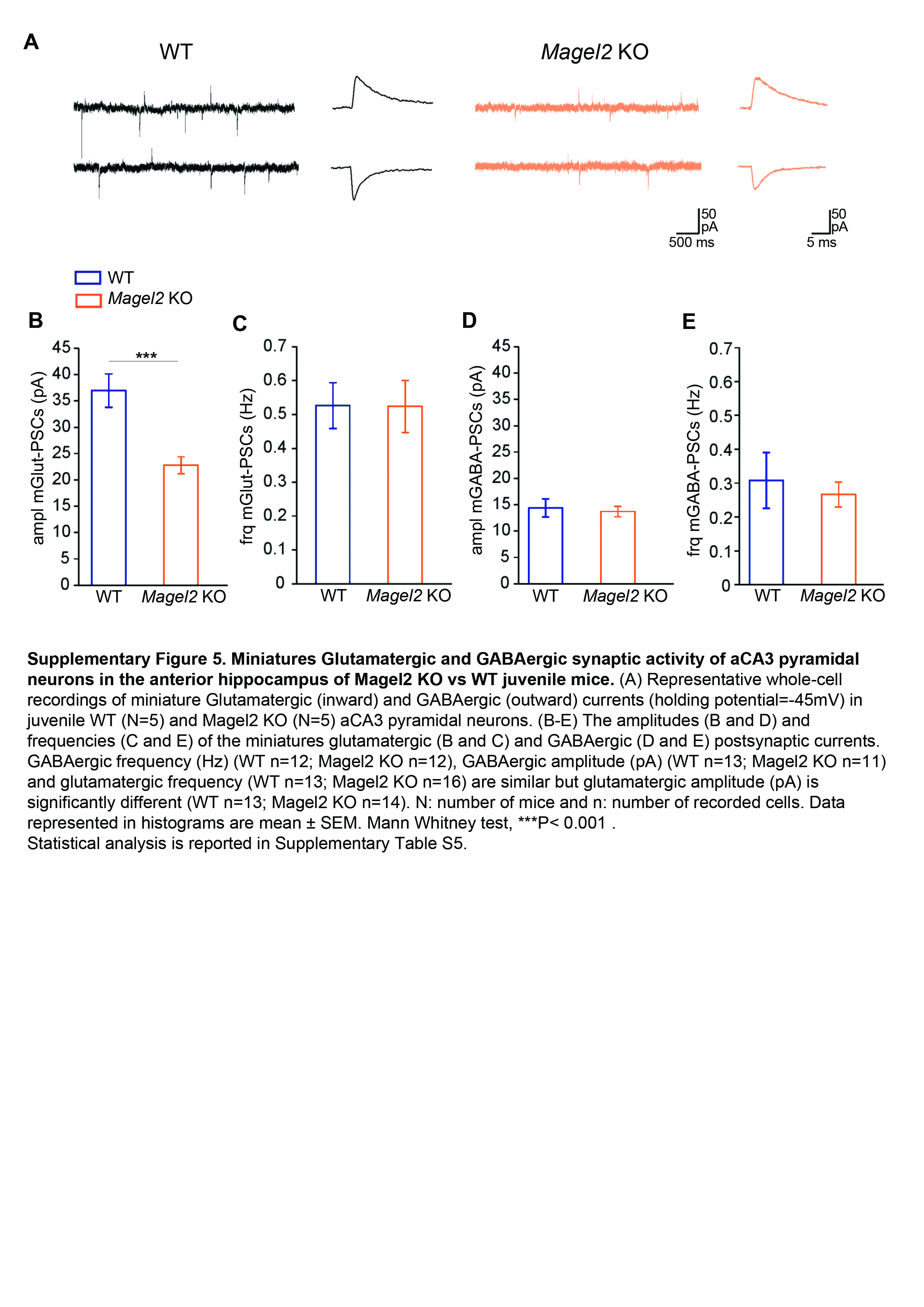

Supplement: Supplementary file 7 — Supplementary Figure 5 [file 41380_2021_1227_MOESM7_ESM.tif]

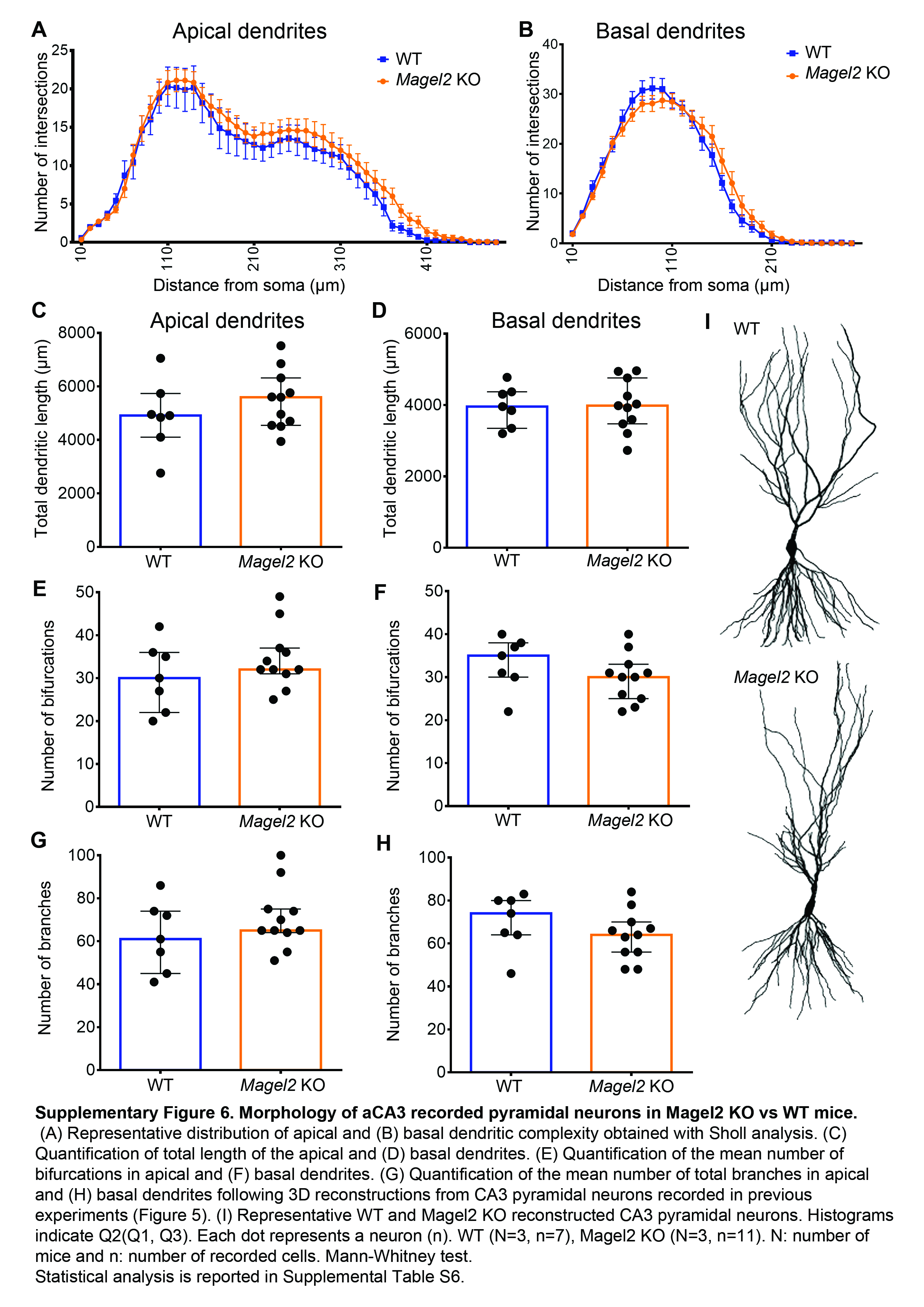

Supplement: Supplementary file 8 — Supplementary Figure 6 [file 41380_2021_1227_MOESM8_ESM.tif]

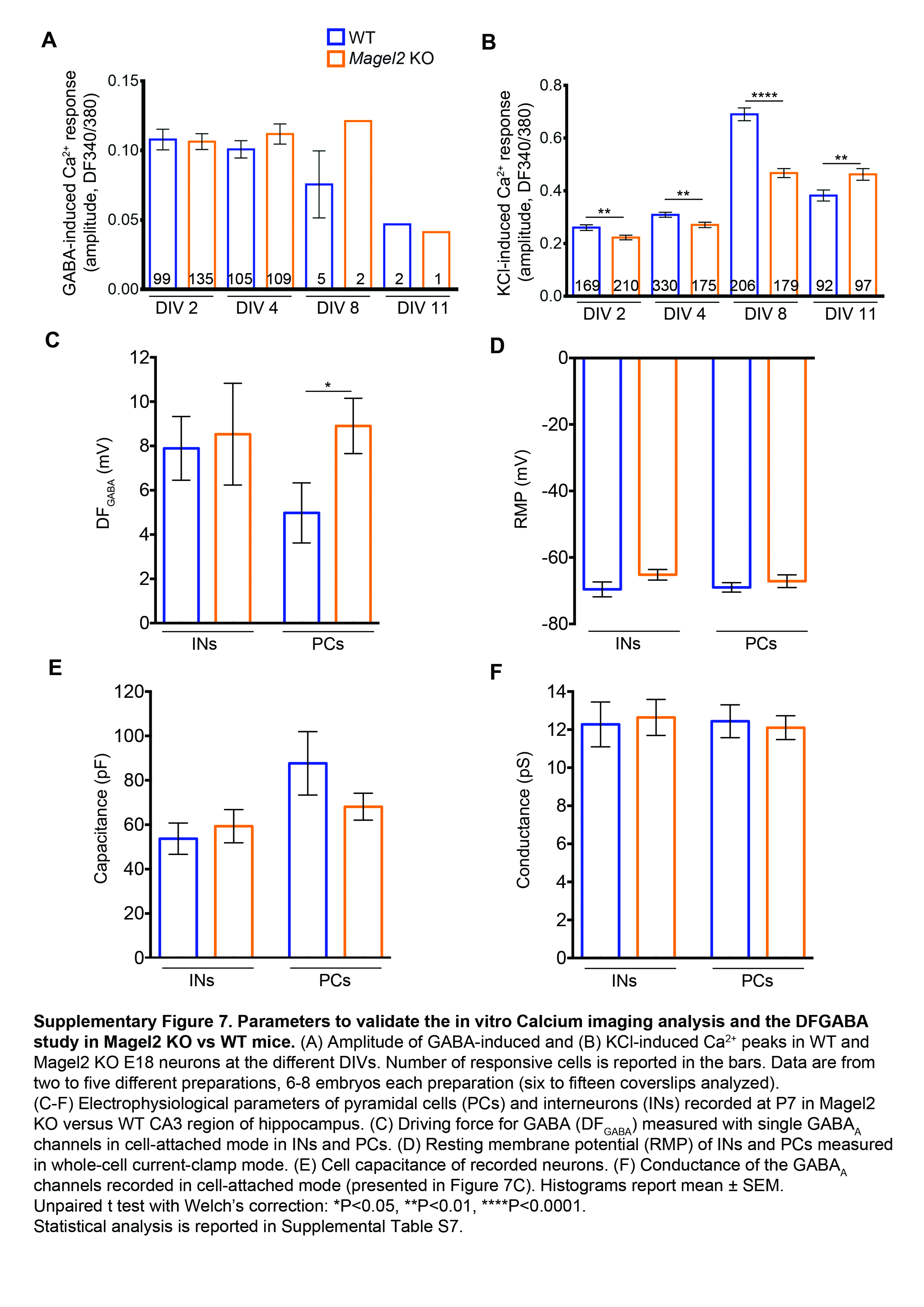

Supplement: Supplementary file 9 — Supplementary Figure 7 [file 41380_2021_1227_MOESM9_ESM.tif]
